# Determinants of Ageism against Older Adults: A Systematic Review

**DOI:** 10.3390/ijerph17072560

**Published:** 2020-04-08

**Authors:** Sibila Marques, João Mariano, Joana Mendonça, Wouter De Tavernier, Moritz Hess, Laura Naegele, Filomena Peixeiro, Daniel Martins

**Affiliations:** 1 Instituto Universitário de Lisboa (ISCTE-IUL), CIS-IUL, 1649-026 Lisboa, Portugal; joao_mariano@iscte-iul.pt (J.M.); joana_mendonca@iscte-iul.pt (J.M.); filomenapeixeiro@campus.ul.pt (F.P.); 2 Center for Social and Cultural Psychology, KU Leuven, 3000 Leuven, Belgium; wouter.detavernier@kuleuven.be; 3 SOCIUM Research Center on Inequality and Social Policy, University of Bremen, 28359 Bremen, Germany; mhess@uni-bremen.de; 4 Department of Ageing and Work, Institute of Gerontology, University of Vechta, 49377 Vechta, Germany; laura.naegele@uni-vechta.de; 5 Department of Neuroimaging, Institute of Psychiatry, Psychology and Neuroscience, King’s College London, London SE58AF, UK; daniel.martins@kcl.ac.uk

**Keywords:** ageism, determinants, systematic review

## Abstract

Ageism is a widespread phenomenon and constitutes a significant threat to older people’s well-being. Identifying the factors contributing to ageism is critical to inform policies that minimise its societal impact. In this systematic review, we gathered and summarised empirical studies exploring the key determinants of ageism against older people for a period of over forty years (1970–2017). A comprehensive search using fourteen databases identified all published records related to the umbrella concept of “ageism”. Reviewers independently screened the final pool to identify all papers focusing on determinants, according to a predefined list of inclusion and exclusion criteria. All relevant information was extracted and summarised following a narrative synthesis approach. A total of 199 papers were included in this review. We identified a total of 14 determinants as robustly associated with ageism. Of these, 13 have an effect on other-directed ageism, and one on self-directed ageism. The quality of contact with older people and the positive or negative presentation of older people to others emerged as the most robust determinants of other-directed ageism; self-directed ageism is mostly determined by older adults’ health status. Given the correlational nature of most studies included in this review, inferences on causality should be made cautiously.

## 1. Introduction

The global population is ageing, and the number of people aged 60 or older is expected to more than double by 2050 [1]. In this demographic scenario, maintaining adequate levels of well-being and health in older people is of crucial importance. Ageism against older people has been widely recognised as a major threat to active ageing and an important public health issue [2]. Several studies have shown that ageistic attitudes, and in particular ageistic stereotypes, have negative impacts on older people in many different domains. In this regard, this work has shown that these negative stereotypes about ageing are acquired at a very early age and tend to act as self-fulfilling prophecies in old age [3,4], leading to poor outcomes for older people in many different areas such as memory and cognitive performance [5], health [6], work performance [7] and even their will-to-live [8]. In addition to this potential negative impact on the individual level, a recent study has also shown that ageism holds important financial costs [9]. 

Ageism is a multifaceted concept including three distinct dimensions: a cognitive (e.g., stereotypes), an affective (e.g., prejudice) and a behavioural dimension (e.g., discrimination). Ageism can operate both consciously (explicitly) and unconsciously (implicitly), and it can be expressed at three different levels: micro-level (individual), meso-level (social networks) and macro-level (institutional and cultural). Furthermore, ageism has two distinct targets [10,11]: On the one hand, ageism can be directed at other individuals—“other-directed ageism”—such as when we think that other older people are slow or wise. On the other hand, ageism can be directed towards oneself—“self-directed ageism” (e.g., I have negative feelings regarding my own ageing).

Ageism is a highly prevalent and widespread phenomenon across many cultures. Data from the World Values Survey [12], including 57 countries, showed that 60% of the respondents reported that older people do not receive the respect they deserve. Across regions, increases in the percentage of older people significantly predicted negative attitudes towards older people [13]. Current trends in global population ageing combined with the absence of directed policies to efficiently address this issue are likely to promote an increase in ageism prevalence over the next decades.

Intervening to reduce ageism and mitigate its harmful impact implies at least some degree of knowledge on the factors contributing or determining its genesis and persistence in our societies. Some theoretical explanations have been put forward by scholars to account for the emergence of negative attitudes toward older people at both societal (e.g., modern societies tend to devalue their older citizens in the sense that they may be perceived as not contributing anymore to the economy [14]) and individual levels (e.g., terror management theory postulates that negative attitudes toward older persons and the ageing process are derived from the fear about our own mortality [15])). Building on these ideas, empirical studies have started to try to identify factors that may contribute to or modulate ageism in different cultural contexts over the last decades. Nevertheless, while evidence has started to emerge, we are still lacking an integrated source of knowledge that allows us to set this research in context and identify which of the factors already explored seem to be more robustly associated with ageism. Thus, we aimed to systematically gather and analyse all available evidence exploring and testing potential explanatory factors for ageism against older people.

To our very best knowledge, current reviews available on the determinants of ageism tend to focus on specific factors and levels of analysis (e.g., cultural context [11], age of the person being evaluated [16]) or follow a literature/critical review format, with no reference to systematic procedures for literature search and analyses (e.g., PRISMA guidelines) [17]. Hence, the present work offers a unique contribution to the field by providing a search, using predefined criteria, of the relevant literature in this area for a vast period of time and offering a synthesis of the main determinants affecting ageism. By giving a comprehensive overview of the main roots of ageism, the paper will allow future research to build on it by, for example, exploring the found determinants in more detail or by closing identified gaps in the literature. In addition, it will be a starting point for policy makers and practitioners (e.g., politicians, employers, teachers) to develop measures to tackle ageism at its roots.

The choice for a systematic review instead of other related methods (such as, a scoping review) [18] was made considering the need to conduct a thorough analysis using clear criteria for paper inclusion and effect evaluation, and to include aspects related to the quality assessment of each study under consideration. In particular, factors such as the design of the studies and sample characteristics seemed fundamental to better explore the causal nature of each determinant and the generalizability of the results obtained. 

Following previous studies aiming to explore determinants in other fields (e.g., [19]), we adopted a socio-ecological perspective using a multi-level framework [20]. This multi-level framework highlights the relevance of both social and environmental factors in shaping human behaviour. More specifically, the following three levels of influence were considered within this framework: intrapersonal, interpersonal/intergroup and institutional/cultural [21]. 

Ultimately, we hope the findings from this systematic review may help to inform the development and expansion of intervention programs aimed at tackling ageism, including the Global Campaign to Combat Ageism that is being led by the World Health Organization (WHO) [22,23,24,25]. In addition, we also aimed to identify and discuss specific research gaps in the determinants of ageism literature where further studies may be beneficial. 

## 2. Materials and Methods 

### 2.1. Literature Search and Eligibility Criteria

A protocol was prospectively developed in accordance with the PRISMA guidelines for Systematic Reviews (see The PRISMA checklist in the Supplementary Materials Table S9). Following current recommendations, the protocol was made openly available through registration with the PROSPERO International Prospective Register of Systematic Reviews platform (www.crd.york.ac.uk/PROSPERO, reference CRD42018089760).

Included studies had the following characteristics: (i) Studies focusing on ageism towards older adults; (ii) Studies aiming to explore determinants of ageism. As determinants, we considered factors that may explain the origins, roots or possible causes of ageism [26,27]; (iii) Studies using an ageism measure as the dependent variable; (iv) Quantitative studies; v) Since the term “ageism” was only introduced in 1969, only studies dated from 1970 onwards were included; (vi) Full text available in English, French, or Spanish. A detailed list of all inclusion and exclusion criteria can be found in Supplementary Table S1.

Specifically, we included studies in which the targets of ageism were 50 years or older. This threshold also allowed us to cover ageism in the labour market, an important area of ageism research, with older workers commonly defined as those aged 50 or older (e.g., Organisation for Economic Co-operation and Development [OECD, [28], and to include studies based on data from ageing surveys, which usually encompass samples of individuals aged 50 or older (e.g., the Health and Retirement Study [HRS] and the Survey of Health, Ageing and Retirement in Europe [SHARE]).

Although our initial protocol included an integrated analysis of both quantitative, qualitative and mixed-method studies, in this review, we decided to focus on findings from quantitative studies. We identified a high number of quantitative (n = 199) and qualitative/mixed methods (n = 90) papers on this topic. Given the high number of quantitative articles identified, the very clear evaluation criteria quantitative studies have to determine whether or not an effect exists, and the extra complexity that would result from including qualitative evidence in the narrative synthesis, we follow the procedure used in other similar studies [29] and only analyse the quantitative evidence at this stage as it represents the majority of the findings of this field, while keeping the number of studies reviewed within a reasonable range. Although we acknowledge this is a limitation regarding our initial goal, these results are still based on a large sample of studies in this area (n = 199) and we believe that they yield meaningful conclusions for research and intervention in this domain. We address this issue in further detail in the discussion section.

### 2.2. Search Strategy and Study Selection

The following electronic databases, including both academic and grey literature, were searched up to 14/12/2017: PubMed, PsycINFO, Ageline, EBSCO, Embase, CINAHL, Global Index Medicus, DARE, Epistemonikos, Cochrane Database of Systematic Reviews, Campbell Collaboration, Prospero, Greylit and Opengrey. A comprehensive search strategy looking at the big umbrella concept of “ageism” was developed for PubMed and then subsequently adapted for the other databases included in the study, as per each database specific thesaurus. The full search string for PubMed can be found in Supplementary Table S2. 

Irrelevant and duplicate studies were initially removed following a two-step process. First, we used a comprehensive deduplication methodology as per by Bramer et al. [30]. Then, the remaining records were imported into COVIDENCE (www.covidence.org), a web-based tool designed to assist the systematic review process, in order to remove additional duplicates and irrelevant records (e.g., articles related to forest “age discrimination” or degenerative diseases related to ageing). Before initiating our screening process, we first piloted our inclusion and exclusion criteria in a sub-sample of 10 references. This pilot was carried out by two independent reviewers (LN, SM). For each of the remaining 13,691 references, title and abstract were independently screened for eligibility by pairs of reviewers selected out of six reviewers (JMa, JMe, LN, MH, SM, WT). Reviewers were randomly assigned to each reference by COVIDENCE (percentage of inter-rater agreement during this process: 92.45%). Disagreements were resolved with the intervention of an additional third randomly assigned reviewer (LN or SM). After this first screening, we identified 647 potentially eligible full texts and uploaded them into COVIDENCE. When full texts could not be found, authors were contacted to provide a copy of the full manuscript (JMe). In the next step, all potentially eligible full texts were examined in detail for eligibility by pairs of reviewers selected out of six reviewers (JMa, JMe, LN, MH, SM, WT). Reviewers were randomly assigned to each reference by COVIDENCE (percentage of inter-rater agreement during this process: 77.55%). Reasons for exclusion were annotated, disagreements resolved with the intervention of a third reviewer (JMa or SM). 

**Figure 1:**
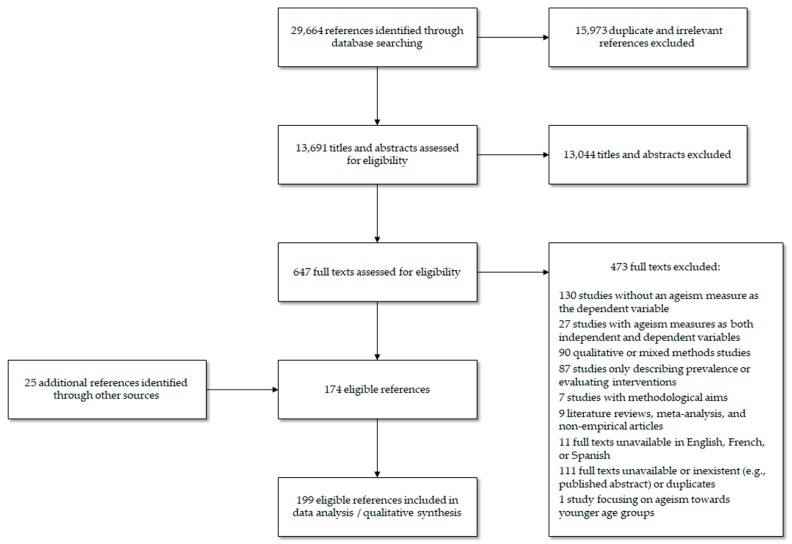
PRISMA flow diagram.

To minimise searching bias, we complemented this approach with a snowball procedure, where we screened all existing reviews/meta-analyses and all references cited in the records we retained after our full eligibility screening. This procedure resulted in the identification of 25 additional relevant records. Figure 1 provides an overview of our search and selection procedures.

### 2.3. Quality Assessment

Given our focus on quantitative studies, we decided to revisit the quality assessment tool initially proposed in our protocol (www.crd.york.ac.uk/PROSPERO, reference CRD42018089760), to be more suitable to the specificities of the type of studies we summarise herein. Each study was then appraised for quality, as per a customised quality assessment tool we developed based on previously validated instruments, namely the Downs and Black checklist [31] and the Newcastle-Ottawa scale [32]. This tool was first piloted in a subset of 10 references (LN, SM), and adjustments were made as necessary. Following the procedure adopted by other authors [33,34], we decided to use a customised appraisal tool to make sure we could capture specific aspects of the methodologies used in this field that, while important, could not be easily captured by a general-purpose instrument. For our purposes, we considered that aspects related with testing of causality and psychometric qualities of the measures used were important, but we could not find any existing measure specifically accounting for these two dimensions together. Moreover, we needed a measure that would consider the heterogeneity in the designs of the studies we reviewed and could be used across a large number of studies. The final version of the tool comprised 11 items addressing the aims and hypotheses of each study, power analysis, participants’ eligibility, description of the methods, adequacy of the methods, testing of causality, psychometric properties of the measures used, ethical considerations, statistical analysis, significance levels and effect sizes. The quality of each of our final list of eligible references was independently assessed by pairs of reviewers randomly selected out of a pool of six reviewers (JMa, JMe, LN, MH, SM, WT), using a three-point scale depending on the level of compliance with each criterion (1 = *low*, 2 = *medium*, 3 = *high*). A global percentage of quality was calculated by dividing the total sum score obtained across items by the total possible score. Percentages of quality were averaged across the two raters to achieve a final quality score. Each study was categorised according to its quality, based on the following criteria: *low:* <60%, *medium:* 60–80%, *high:* >80%. No study was discarded due to poor quality.

### 2.4. Data Analyses

#### 2.4.1. Extraction

The data extraction form was piloted together with the quality assessment tool (LN, SM) and adjustments were made as necessary. The final extraction form included entries on: publication details (e.g., year, country, format), research method (e.g., participants, design, procedure), ageism outcome (e.g., definition, measure, classification), and determinants explored (e.g., definition, measure, effect significance and direction). For each included reference, one reviewer extracted all relevant data for all entries in the form. Following current gold-standard procedures for systematic reviews and meta-analyses [35], a second reviewer independently extracted critical information for entries related to determinants and confirmed the data extracted by the first reviewer. Disagreements and inconsistencies were resolved with the intervention of a third reviewer (FP or SM).

#### 2.4.2. Synthesis

Given the wide-range and high heterogeneity of the studies included in this systematic review, we summarised our findings using a narrative synthesis procedure. Taking into consideration the different levels where ageism takes expression and following similar endeavours in other fields [20,21,26], we categorised each determinant according to a multi-level framework where we considered individual, interpersonal/intergroup and institutional/cultural levels of expression.

In our main synthesis analysis, only determinants studied in at least three papers were taken into account [29] (see Supplementary Material Tables S4 and S6 for the complete list of determinants considered in more than three papers; for determinants considered in less than three papers, see Tables S5 and S7 of the Supplementary Materials). For each determinant, papers were organised in one of three categories: finds a positive effect; finds a negative effect; and non-significant/mixed association (ns/mix) with ageism. The latter includes studies finding no significant relationship (significance threshold of *p* < 0.05), and studies analysing multiple dependent variables as aspects of ageism, for which the effects of the determinant were not consistent across dependent variables. This procedure has been adapted from previous studies exploring determinants using a multiple levels of analysis approach, such as the one we used herein [29]. Following previous studies [19], we considered a determinant to be robust if at least 60% of the studies where the determinant was examined agree on both the existence and the direction of the effect of the determinant.

## 3. Results

**Figure 2:**
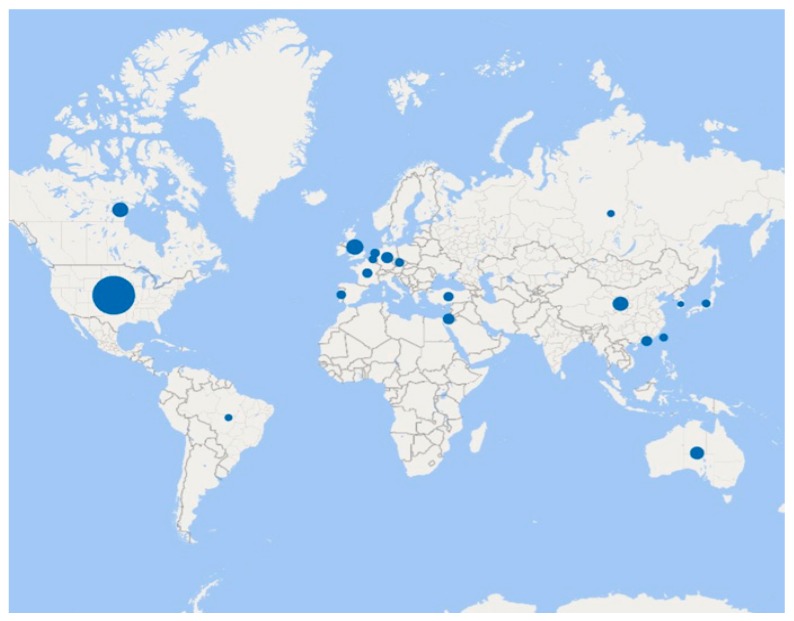
Main geographic distribution of the studies included in this review.

A total of 199 papers were included in this review. Most papers collected samples from western countries, particularly from the United States of America (n = 119; 59.80%), and more than two in five papers were published since 2010 (n = 85; 42.71%) (see Figure 2 for a visual representation of the main distribution of studies per country, and for more detailed information see Table S3 in the Supplementary Materials). 

For most studies, more than half of participants were female (n = 121; 60.80%) and included participants under 50 years of age (n = 105; 52.76%) or both below and over 50 years old (n = 65; 32.66%) (Table 1). Most studies were cross-sectional (n = 123; 61.81%) and measured at least the cognitive dimension of ageism (n = 185; 92.96%) and the majority in an explicit manner (n = 192; 96.48%) (a complete overview of measures of ageism used is presented in Supplementary Materials Table S3). 

Eighty-eight studies scored “High”, 107 studies scored “Medium” and four studies scored “Low” in our quality assessment analyses. An overview of each study’s compliance with our criteria is presented in Figure S1 in the Supplementary Materials. A score on the quality of evidence per study is presented in Table S8 in the Supplementary Materials. The main strengths of the studies included were the clear description of aims and study procedures, methodological and statistical approaches and the high psychometric quality of the measures included. Studies were limited in relation to the possibility of testing for causal relationships, in including a priori calculation sample sizes and in the lack of clarity and/or detail in describing ethical procedures or the eligibility criteria for participants.

The large majority of the studies included in this review examined other-directed ageism, meaning stereotyping of, prejudice against or discrimination of other individuals based on their age (n = 179; 89.95%). Only a small number of studies focused on self-directed ageism (n = 11; 5.53%) or ageism directed at both other and self (n = 9; 4.52%). Determinants contributing to both other and self-directed ageism were included both in the other and the self-directed ageism analyses.

### 3.1. Other-Directed Determinants of Ageism

We identified a total of 31 determinants that were examined in at least three articles regarding other-directed ageism, including 20 at the individual level, nine at interpersonal/intergroup level, and two at institutional/cultural level (Table 2). We present below a detailed description of the determinants found for each of our levels of analysis in separate sub-sections.

#### 3.1.1. Intrapersonal Level Determinants

Some of the most solid findings regarding intrapersonal-level determinants of other-directed ageism concern *behavioural and psychological factors*. Eight out of nine papers found that “anxiety of ageing” increases ageism in the individual, and seven out of nine papers also found a positive association with “fear of death”. Personality traits such as consciousness (two out of three), agreeableness (three out of three), extraversion (two out of three) and having a collectivistic orientation (two papers) were found to be associated with a decrease in other-directed ageism.

Age (81 papers) and sex (67 papers) of the respondents were the two individual-level determinants most commonly explored in the papers included in this review. However, the majority of studies did not find a (consistent) age or sex effect. The evidence is inconclusive about the effects of other sociodemographic characteristics, including years of education (24 papers), cultural background (18 papers), ethnicity (13 papers), socio-economic status (six papers), religiosity (five papers), living in an urban versus rural area (five papers) and marital status (three papers). Studies were also inconclusive regarding the effects of health status (six papers) and of activity-related determinants (six papers on professional experience and seven on studying ageing and care-related topics).

#### 3.1.2. Interpersonal/Intergroup Level Determinants

The evidence is inconclusive about whether the frequency of contact between younger and older individuals reduces ageism on the subject (29 papers). However, 10 out of 13 papers found that the quality of this contact does reduce the prevalence of ageism. When asked specifically about contact with grandparents, results follow a similar pattern: whereas 7 out of 10 papers show a robust association of the quality of contact with grandparents with ageism, the results are mixed regarding the effect of quantity of contact with grandparents (10 out of 18 papers). The characteristics of older targets presented in the studies also seem to matter. Whereas studies are inconclusive about whether female targets are more likely to be targets of higher ageism (21 studies), 17 out of 27 papers did find that stereotypes are more likely to emerge if the age of the target is higher. Furthermore, the frame under which the older individual is presented seems highly relevant: all 13 papers in which the older target was presented in a positive way found that this positive presentation reduced ageism, whereas 13 out of 14 papers where the target was presented in a negative way found that this presentation amplified ageism. In relation to activity—including respondents’ experience of caregiving or working with older people—there were mixed findings (four out of eight papers).

#### 3.1.3. Institutional/Cultural Level Determinants

Only a few studies examined determinants of ageism at this level. We found only two robust determinants at this level: available societal economic resources (three out of five papers) and percentage of older people in the country (two out of three papers).

### 3.2. Other-Directed Determinants of Ageism: Differences by Participants Age Group

A sub-group analysis considering only the robust “other-directed” determinants of ageism by age group highlighted that a much higher number of articles relied on younger participants (n = 104) than on older participants (n = 36) (Table 3). The pattern of results for younger participants follows, in general, the one identified for the whole sample of studies. However, in the case of older participants, only few determinants were identified as being robustly associated with ageism. These determinants generally followed the same direction identified in the analysis including the whole sample of studies and were: anxiety about ageing (three out of three papers), fear and salience of death (four out of four papers), target’s age (seven out of 10 papers), older persons presented negatively (three out of three papers), older persons presented positively (three out of three papers) and available economic resources (two out of three papers). The remaining determinants did not reach our threshold for being considered in the analyses (in the sense that there are less than three papers exploring that specific determinant for this age group). It is important to highlight that some papers did not provide a complete description of the sampling procedure or did not involve the contribution of human participants (e.g., analyses of content in the media). Therefore, these cases were not considered in this further analysis (e.g., the study by Ng et al. [169] explores the percentage of older persons in the country considering a method of computational linguistic analysis to the corpus of Historical American English).

### 3.3. Self-Directed Forms of Ageism

Table 4 shows the nine determinants we identified as being explored in at least three articles regarding self-directed forms of ageism. Similarly to what happened for other-directed ageism, most intrapersonal-level determinants examined showed no relevant association. The only exception was mental and physical health status (eight out of nine papers), which was found to be associated with lower levels of self-directed ageism. It is interesting to note that this determinant was not a significant predictor of other-directed forms of ageism.

Finally, no robust determinants of self-directed ageism were found at the interpersonal/intergroup and institutional/cultural levels of analysis.

**Figure 3:**
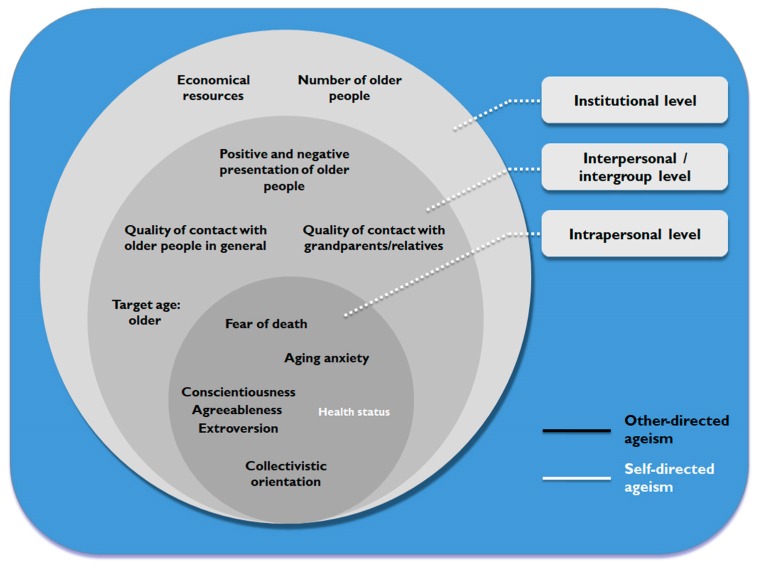
Determinants of ageism across intrapersonal, interpersonal/intergroup and institutional levels.

Figure 3 presents a visual representation of the main determinants of ageism identified in this review (other and self-directed) at the intrapersonal, interpersonal/intergroup and institutional levels.

## 4. Discussion

In this manuscript, we present the results of the first systematic overview on determinants of ageism against older people. We mapped and summarised evidence exploring determinants of ageism against older people in virtually all quantitative studies conducted for over a forty-year period. We identified which of the determinants explored present a more robust association with ageism and, therefore, should constitute priorities in policies of interventions aiming to fight ageism against older people. We categorised all determinants we found to be robustly associated with ageism using a multi-level framework [20], which considered sources of influence from individual, interpersonal/intergroup and institutional/cultural levels for both other and self-directed forms of ageism. Our findings come with important implications for the development and expansion of current policies against ageism, as discussed below.

Different sets of determinants seem to contribute to other and self-directed forms of ageism. Studies on other-directed determinants have mainly focused on the effect of intrapersonal-level determinants. Here, the most robust determinants are individuals’ “anxiety of ageing” and “fear of death”. From a pre-emptive perspective, one may argue that the impact of “fear of death” may be difficult to reduce, as terror management theories postulate that this fear is deep-seated, and even fundamental to the human condition [234]. However, at the same time, our study also suggests that educational efforts to address the representations of illness and death hold the potential to change how contemporary societies perceive and understand ageing [235]. At the individual level, studies have also shown that specific personality traits (e.g., conscientiousness and agreeableness) and individual psychological factors (e.g., personal degree of collectivistic orientation) work to mitigate ageism against older people. This finding is in tune with personality-based theories of prejudice [236] and highlights the need to consider intra-individual differences when designing and implementing interventions to pre-empt ageism.

At the interpersonal and intergroup levels, contact with older people seems to be the most important determinant of other-directed ageism. It is commonly accepted that contact with older individuals in itself is sufficient to reduce ageism—the fact that we identified more than twice as many studies dealing with the effect of contact frequency as compared to contact quality supports this general belief. However, our findings point to the importance of the quality of the contact over frequency and to the importance of how older individuals are presented (we are less likely to stereotype older individuals of whom we have a positive image). Therefore, one can hypothesise that ageism could be reduced by stimulating intergenerational contact in a positive context—this may include, for instance, promoting initiatives where younger individuals may work with older individuals and share experiences. Following the same rationale, attention should also be directed at the portrayal of older people and ageing in media content, where the presentation of more positive images of older adults offers a promising avenue to tackle ageism [237].

At the institutional and cultural level, only two determinants were identified as robustly associated with other-direct ageism: the availability of resources in society and the percentage of older people in the country. As scarcity of resources increases, especially in the face of an increase in the number of older people [13], tensions over resource allocation tend to spark, leading to higher rates of ageism. It is, therefore, reasonable to assume that ageism will decrease as societies develop economically [223].

In self-directed ageism, only intrapersonal determinants have been thoroughly explored. However, out of the nine factors identified, only individuals’ mental and physical health showed a robust association. This result is important because it highlights the need to invest in policies promoting active and healthy ageing practices that allow individuals to live longer, healthier and happier lives [238]. We could not find any robust association between self-directed forms of ageism and determinants at the interpersonal/intergroup and institutional/cultural levels of analysis.

### Limitations and Recommendations for Future Studies

Whereas some factors have been widely studied without consensus on their effect on ageism, such as age and sex, others have been largely ignored. At the individual level, we found very few studies dealing, for instance, with individuals’ norms, age group identification and cognitive processes. Moreover, the role of institutional and cultural factors (e.g., age discrimination laws) in the development and expression of ageism still remains a blind spot when we consider the literature altogether. Future research clarifying whether these factors may play a role will be more than welcome given their important policy implications (e.g., anti-age discrimination legislation). It would also be important to invest in exploring further factors that yielded inconclusive results so far. For instance, despite the idea that older women may be perceived as per a “double-standard” [63] of ageing—being rated more negatively than men—we could not find a consistent effect of gender of the target being evaluated. In fact, some studies show that this effect does not seem to occur for all measures of ageism and/or in all domains. For instance, Kornadt and colleagues [145] found that women were rated more positively than men in domains such as friendship, leisure and health; however, they were rated worse than men in the domains of finances and work. In the same vein, it would also be interesting to expand research to explore neglected factors, such as self-related aspects and other-directed forms of ageism expressed by older people themselves. Taking into consideration that there is a vast body of research showing that older people are especially prone to be affected by ageism and self-stereotypes [239,240], it would be important to deepen our knowledge about which factors, beyond health-related aspects, may influence self-directed ageism.

One major drawback of our work is the fact that our results derive mainly from studies conducted in English-speaking countries (e.g., USA) and with female, young participants. This aspect raises questions about the generalisability of our findings to other contexts where ageism prevails [12]. Furthermore, in this manuscript we report only on quantitative studies, leaving aside qualitative evidence. We believe that also taking this research into consideration would be fruitful in the future in the sense that qualitative studies offer the opportunity for rich and in-depth detail, which may be advantageous in situations where a detailed understanding may be required, such as understanding how these determinants can contribute to ageism. We also found that studies in this field of research are mostly correlational in nature, which limits inferences on the causal contribution of the determinants identified herein to ageism (e.g., fear of death may be caused by ageism itself). Future research capitalising on experimental designs may address this limitation, at least for some of the determinants presented herein.

In this work, we adopted a narrative synthesis where we analysed: i) the number of papers that explored a certain determinant; ii) within these papers, how many found a significant association of the determinant with ageism (where we classified the direction of the effect as “positive” or “negative” or “non-significant or mixed”). The strength of the relationship of the determinant with ageism is thus given by the percentage of papers that found a significant relationship, within the ones where that specific determinant was explored. As per previous literature on the study of determinants [19] in other fields, we took a semi-quantitative approach according to which we considered a determinant to be robustly associated with ageism if there was a significant relationship in a consistent direction in at least 60% of the papers that explored this determinant. While we decided to keep our scope broad and not to conduct quantitative synthesis on specific determinants, we are convinced that our findings help to identify specific determinants for which meta-analyses may be feasible and worth investigating.

Finally, it is important to consider that this work was developed in the context of a large-scale and collaborative effort to identify, summarise and synthesise virtually all relevant literature in this field of research, over a large period of time. However, despite all efforts implemented to minimise searching bias (i.e., snowballing procedure), it is possible that relevant papers were missed. Also, although our study spanned a large period of time, including virtually all papers from 1970 to 2017 (and also online versions of published papers in 2018), we did not include all papers potentially published since the end-date of our searching period until now. Our review departed from a vast pool of studies, which allowed us to minimise searching bias but delayed time from searching to dissemination. We decided to not conduct an immediate update to keep our strategy consistent with the remainder of projects developed in the context of the same initiative—the Global Campaign to Combat Ageism of the WHO [22,23,24,25]. While our work is the first systematic review effort on this topic and sets bases for future endeavours within the same scope, we acknowledge it may be important to revisit this topic in a few years for an update (may further developments in the field justify).

## 5. Conclusions

Ageism is one of the major threats to active ageing and manifests itself on a range of domains from individual to institutional and cultural levels [2]. Tackling ageism should be a priority for policy makers, and it seems obvious from our findings that a campaign to combat ageism will necessarily need to consider factors spanning different levels/domains in order to be successful. We believe our review will support these efforts by helping to identify major factors that have been empirically and robustly demonstrated to contribute to negative visions of ageing and older people. At the same time, we also hope this work may entice further research bridging the research gaps our integrated appraisal of the literature highlighted.

## Figures and Tables

**Table 1:** Characteristics of the studies included in this review.

First Author	Year	Target	Age	Sex	Design	I-P	D-Ageism
Adams-Price [36]	2009	OT	Y,O	F	Exp	E	C
Allan [37]	2014	OT	Y	F	Cros	E	C,A,B
Ayalon [38]	2013	OT	Y,O	F,M	Cros	E	A
Ayalon [39]	2016	S	O	F	Long	E	B
Bacanli [40]	1994	OT	Y	F	Cros	E	C
Baker [41]	1983	OT	Y	F	Cros	E	C
Beatty [42]	2009	OT	Y	F	Cros	E	C
Beck [43]	1979	OT	Y	--	Exp	E	C
Bell [44]	1973	OT	Y,O	F	Exp	E	C
Bergman [45]	2013	OT	Y	F	Cros	E	C,A,B
Bhana [46]	1983	OT	Y	F,M	Exp	E	C
Bieman-Copland [47]	2001	OT	Y,O	F	Exp	E	C
Bierly [48]	1985	OT	Y	F	Cros	E	C
Bodner [49]	2014	OT	Y	F	Cros	E	C,A,B
Bodner [50]	2010	OT	O	F	Cros	E	C,A,B
Bodner [51]	2008	OT	Y	F	Cros	E	C,A,B
Bodner [52]	2011	OT	O	F	Cros	E	C,A,B
Bodner [53]	2012	OT	Y,O	F	Cros	E	C,A,B
Bodner [54]	2015	OT	O	F	Cros	E	C,A,B
Boudjemadi [55]	2012	OT	Y	F	Exp	I	A
Bousfield [56]	2010	OT	Y	M	Cros	E	C,A,B
Bowen [57]	2013	OT	Y,O	F,M	Cros	E	C
Braithwaite [58]	1986	OT	Y	F	Exp	E	C
Braithwaite [59]	1993	OT	Y	F	Long	E	C,A
Brewer [60]	1984	OT	O	F	Exp	I	C
Bryant [61]	2014	S	O	F	Long	E	C,A,B
Burge [62]	1978	OT	Y,O	F	Cros	E	C
Canetto [63]	1995	OT	Y,O	F	Exp	E	C
Cary [64]	2013	OT	Y,O	F	Exp	E	C,A
Caspi [65]	1984	OT	Y	F	Cros	E	C
Celejewski [66]	1998	OT,S	Y,O	F	Exp	E	C
Chan [67]	2012	OT	Y	F	Cros	E	C
Chang [68]	1984	OT	Y	M	Cros	E	C
Chasteen [69]	2000	OT, S	Y,O	F	Exp	E	C
Chasteen [70]	2005	OT, S	Y,O	F	Exp	E	C,B
Chen [71]	2010	OT	Y,O	F	Exp	E	C
Chen [72]	2017	OT	Y	F	Exp	E	C
Cherry [73]	2015	OT	Y,O	F	Cros	E	C,A,B
Cheung [74]	1999	OT	Y,O	F	Cros	E	C
Cheung [75]	2011	OT	Y,O	F	Cros	E	C,B
Chiu [76]	2001	OT	Y,O	F	Cros	E	C,B
Choi [77]	2013	OT	Y	M	Exp	E	C,B
Chonody [15]	2016	OT	Y,O	F	Cros	E	C,A,B,
Chopik [78]	2017	OT	Y,O	F	Cros	E, I	C,A,B,
Chou [79]	2011	S	O	F	Cros	E	B
Chung [80]	2012	OT	Y,O	F	Cros	E	C
Clément-Guillotin [81]	2015	OT	Y	M	Exp	E	C,A,B,
Collette-Pratt [82]	1976	OT	Y,O	F	Cros	E	A
Connor et al. [83]	1978	OT	Y	F,M	Exp	E	C,B
Cox [84]	2012	OT	Y	M	Exp	E	C,B
Crew [85]	1984	OT	Y	M	Cros	E	C
Cullen [86]	2009	OT	Y	F	Exp	E, I	C,A
DaŞBaŞ [87]	2015	OT	Y,O	F	Cros	E	C
Dasgupta [88]	2001	OT	Y	F	Exp	E,I	C,A
Davidson [89]	2008	OT	Y	F,M	Cros, Exp	E	C,B
DeGuzman [90]	2014	S	O	F	Cros	E	B
Demir [91]	2016	OT	Y	F	Cros	E	C
Depaola [92]	1992	OT	--	F	Cros	E	C,A
Depaola [93]	1994	OT	--	F	Cros	E	C,A
Depaola [94]	2003	OT	O	F	Cros	E	C
dePaulaCouto [95]	2012	OT	Y,O	F	Cros	E	C
Deuisch [96]	1986	OT	Y,O	F,M	Exp	E	C
Diekman [97]	2007	OT	Y	F	Exp	E	C,B
Donlon [98]	2005	OT	0	F	Cros	E	C
Drury [99]	2016	OT	Y	F	Cros	E	C,A
Drydakis [100]	2018	OT	Y,O	--	Exp	I	B
Duncan [101]	2009	OT	Y	--	Exp	I	C,A
Faulkner [102]	2007	OT	Y	F	Exp	E	C
Ferraro [103]	1992	OT	Y,O	--	Rep Cros	E	C
Finkelstein [104]	1998	OT	Y,O	M	Exp	E, I	C
Folwell [105]	1997	OT	Y	M	Exp	E	C
Freeman [106]	2002	OT	Y	F	Cros	E	C
Fullen [107]	2016	S	O	F	Cros	E	C
Fusilier [108]	1983	OT	Y	M	Exp	E	C
Gattuso [109]	1998	S	Y	F	Cros	E	C,A,B
Gattuso [110]	2002	OT	Y	F	Cros	E	C,A,B
Gekoski [111]	1990	OT	Y	F,M	Exp	E	C
Gekoski [112]	1984	OT	Y	F,M	Exp	E	C
Gibson [113]	1993	OT	Y,O	--	Exp	E	C
Gluth [114]	2010	OT	Y,O	M	Cros	E	C
Gordon [115]	1988	OT	Y,O	F,M	Exp	E, I	C,B
Graham [116]	1989	OT	O	--	Cros	E	C
Hale [117]	1998	OT	Y,O	--	Cros	E	C
Harris [118]	1988	OT	Y	M	Cros	E	C
Harwood [119]	1994	OT	Y	F	Exp	E	C
Harwood [120]	2001	OT	O	F	Cros	E	C
Harwood [121]	2005	OT	Y	F	Cros	E	C,A
Haught [122]	1999	OT	Y	--	Cros, Cohort	E	C
Hawkins [123]	1996	OT	Y	F	Cros	E	C
Hehman [124]	2012	OT	Y	F,M	Exp	I	B
Hertzman [125]	2016	OT	Y	F	Cros	E	C,B
Huang [126]	2013	OT	Y	F	Cros	E	C
Hughes [127]	2016	OT	Y	F,M	Cros	E	C,A,B
Hummert [128]	1997	OT	Y,O	F	Exp	E	C
Hummert [129]	2002	OT	Y,O	F	Exp	E,I	A
Hummert [130]	1993	OT	O	--	Cros	E	C
Hummert [131]	1994	OT	Y	--	Cros	E	C
Iweins [132]	2012	OT	Y,O	F	Cros,Exp	E	C,A,B
Jackson [133]	1988	OT	Y,O	F	Cros	E	C
Janečková [134]	2013	OT, S	O	F	Cros	E	C,A,B,
John [135]	2013	OT	Y,O	F,M	Cros	E	C
Kalavar [136]	2001	OT	Y	F	Cros	E	C,A,B,
Kane [137]	2006	OT	Y	F	Cros	E	C
Karpinska [138]	2011	OT	Y	M	Exp	E	B
Katz [139]	1990	OT	Y,O	F	Cros	E	C,A
Kirk [140]	2015	OT	Y,O	F	Cros	E	C,A,B,
Knox [141]	1989	OT	Y	F,M	Quasi-Exp	E	C
Knox [142]	1986	OT	Y	F,M	Cros	E	C
Kornadt [143]	2017	OT	Y,O	F	Cros	E	C
Kornadt [144]	2011	OT	Y,O	F,M	Cros	E	C
Kornadt [145]	2013	OT	Y,O	F	Cros	E	C
Krendl [146]	2016	OT	Y	F,M	Cros	E	C,A
Kuhlmann [147]	2017	OT	Y,O	F	Exp	E	C
Kulik [148]	2000	OT	Y	F	Exp	E	C,B
Kwong See [149]	2009	OT	Y	M	Exp	I	C
Laditka [150]	2011	OT	Y	F,M	Cros	E	C,A,B
Laidlaw [151]	2010	S	O	F	Cros	E	C,A,B
Lamont [152]	2017	S	O	F	Cros	E	C,A
Levy [153]	1999	OT, S	Y,O	F	Cros	E	C
Levy [154]	2008	OT, S	O	M	Long	E	C,A
Levy [155]	2015	OT	O	F	Long	E	C
Lin [156]	2009	OT	Y	F	Cros	E	C,A,B
Linville [157]	1982	OT	Y	M	Exp	E	C,A
Locke-Connor [158]	1980	OT	Y,O	F	Exp	E	C,B
Löckenhoff [159]	2009	OT	Y	F	Cros	E	C
Lookinland [160]	1995	OT	Y,O	F	Cros	E	C
Luchesi [161]	2016	OT	O	F	Cros	E	C
Luo [162]	2013	OT	Y	F	Cros	E	C,A,B
Luszcz [163]	1986	OT, S	Y,O	F	Cros	E	C
Lytle [164]	2016	OT	Y	F	Exp	E	C,A,B
Marquet [165]	2016	OT	Y	M	Cros	E	C,A,B
Martens [166]	2004	OT	Y	F	Exp	E	C,A
McCann [167]	2013	OT	Y	F	Cros	E	C
McNamara [168]	2016	OT	Y,O	F	Cros	E	C
Melanson [169]	1985	OT	Y	--	Cros	E	C
Miller [170]	1984	OT	Y	M	Exp	E	C,B
Milligan [171]	1985	OT, S	O	M	Cros	E	C
Milligan [172]	1989	OT	Y,O	F	Exp	E	C
Montepare [173]	1988	OT	Y	F,M	Exp	E	C
Narayan [174]	2008	OT	Y	M	Exp	E	C
Ng [175]	2015	OT	NA	NA	Cros	E	C
Nochajski [176]	2011	OT	Y	M	Cros	E	C
Nochajski [177]	2009	OT	Y	M	Long	E	C
North [178]	2013	OT	Y,O	F	Exp	E	C,B
North [179]	2016	OT	Y,O	M	Exp	E	C,B
O’Connell [180]	1979	OT	Y	F,M	Exp	E	C
O’Connor [181]	2012	OT	Y	F	Exp	E	C,A
Obhi [182]	2016	OT	Y	F	Cros	E	C,B
Okoye [183]	2005	OT	Y	F	Cros	E	C
Oliveira [184]	2015	OT	Y	F,M	Cros	E	C
Özdemir [185]	2016	OT	Y	--	Cros	E	C,B
Paris [186]	1997	OT	Y	M	Cros	E	C
Passuth & Cook (1985) [187]	1985	OT	Y,O	--	Cros	E	C
Pecchioni [188]	2002	OT	Y	F	Cros	E	C
Randler [189]	2014	OT	Y	F,M	Cros	E	C
Reed [190]	1992	OT	Y	--	Cros	E	C
Revenson [191]	1989	OT	Y,O	M	Exp	E	C
Rittenour [192]	2016	OT	Y	F	Exp	E	C,A
Roberts [193]	2008	OT	--	F	Cros	E	C
Robertson [194]	2017	OT	Y,O	F	Cros, Exp	E	C
Ruiz [195]	2015	OT	Y	F,M	Cros	E, I	C,A,B
Runkawatt [196]	2013	OT	Y,O	--	Cros	E	C
Ruscher [197]	2000	OT	Y	F	Cros	E	C
Ryan [198]	2004	OT	Y,O	F,M	Cros	E	C
Ryan [199]	1990	OT	Y	F	Exp	E	C,B
Sanders [200]	1987	OT	Y	F	Quasi-Exp	E	C
Sargent-Cox [201]	2012	S	O	M	Long	E	C,A
Sheier [202]	1978	OT	Y	F	Exp	E	C
Schwartz [203]	2001	OT	Y	F	Cros	I	C
Sherman [204]	1978	OT	Y,O	F	Cros	E	C
Sherman [205]	1985	OT	O	F	Cros	E	C,B
Signori [206]	1982	OT	Y,O	F	Cros	E	C
Skorinko [207]	2013	OT	Y	F	Exp	E,I	C
Smith [208]	2017	OT	Y	F	Cros	E	C
Soliz [209]	2003	OT	Y	F	Cros	E	C,A
Solomon [210]	1979	OT	Y	--	Cros	E	C
Springer [211]	2015	OT	Y	F	Exp	E	C
Steitz [212]	1987	OT	Y	M	Long	E	C
Stewart [213]	2005	OT	Y	F	Cros	E	C
Stewart [214]	1982	OT	Y	M	Exp	E	C
Stier [215]	1980	OT	Y	F,M	Exp	E	C
Stokes [216]	2016	S	O	F	Cros	E	B
Tam [217]	2006	OT	Y	F	Cros	E,I	A
Tan [218]	2004	OT	Y	F	Cros	E	C
Thorson [219]	1974	OT	Y,O	--	Cros	E	C
Tomko [220]	2013	OT	Y,O	F	Cros	E	C
Trigg [221]	2012	OT,S	O	M	Cros	E	C,A,B
Turner [222]	2010	OT	Y	F	Exp	E,I	A
Vauclair [223]	2015	OT	Y,O	F,M	Cros	E	C
Vauclair [224]	2017	OT	Y	M	Cros	E	C,A,B
Vauclair [225]	2017	OT	Y	F,M	Cros	E	C, A
Verhaeghen [226]	2011	OT	Y	M	Exp	E	C
Vrugt [227]	1996	OT	Y	--	Exp	E	C
Waldrop [228]	2003	OT	Y,O	F	Cros	E	C
Wang [229]	2009	OT	Y	F,M	Cros	E	C
Wingard [230]	1982	OT	Y,O	F	Exp	E	C
Wurm [231]	2014	S	Y,O	F,M	Cross	E	C,A
Zhang [232]	2016	OT	Y,O	F,M	Cros, Exp	E	C
Zweibel [233]	1993	OT	Y,O	F	Exp	E,I	B

Note: Year—Year of study publication; Target—Target of ageism; Age—Age of participants in the studies; Sex—Sex of participants in the studies; Design—Study design; I-P—Implicit or explicit measure of ageism; D-Ageism—Dimension of ageism; OT—Other-directed ageism; S—Self-directed ageism; Y—Majority of younger participants; O—Majority of older participants; YO—Both younger and older participants; F—Majority of female participants; M—Majority of male participants; F, M—Both male and female participants; Cross—Cross-sectional design; Exp—Experimental design; Long—Longitudinal design; Rep Cross—repeated cross-sectional design; Quasi-exp—Quasi-experimental design; E—Explicit measures of ageism; I—Implicit measures of ageism; C—Cognitive dimension of ageism; A—Affective dimension of ageism; B—Behavioural dimension of ageism.

**Table 2:** Determinants of “other-directed forms of ageism” (total N = 188).

	Number Overall Papers (n ≥ 3)	Direction of the Association with Ageism (n; %)			Assoc (+, −, ns/mix)
		Pos	Neg	ns/mix	
**Intrapersonal level**					
Demographics (participants)					
Age (older)	81	8 (9.88)	32 (39.50)	41 (50.62)	ns/mix
Sex (being a male)	67	23 (34.32)	3 (4.47)	41 (61.19)	ns/mix
Years of education	24	2 (8.33)	7 (29.17)	15 (62.50)	ns/mix
Cultural background (East vs. West)	18	4 (22.22)	1 (5.56)	13 (72.22)	ns/mix
Ethnicity: Black vs. White	13	5 (38.46)	0 (0)	8 (61.53)	ns/mix
Ethnicity: Lat/Hisp vs. White	7	2 (28.57)	0 (0)	5 (71.42)	ns/mix
Ethnicity: Asian vs. White	6	0 (0)	0 (0)	6 (100)	ns/mix
Study area: ageing & care	7	1 (14.28)	2 (28.57)	4 (57.14)	ns/mix
Professional experience	6	0 (0)	3 (50)	3 (50)	ns/mix
Better physical and mental health condition	6	0 (0)	1 (16.67)	5 (83.33)	ns/mix
Socio-economic status	6	0 (0)	0 (0)	6 (100)	ns/mix
Degree of religiosity	5	0 (0)	2 (40)	3 (60)	ns/mix
Living in Urban vs. Rural	5	2 (40)	0 (0)	3 (60)	ns/mix
Marital status (being married)	3	0 (0)	1 (33.33)	2 (66.66)	ns/mix
Behavioural and psychosocial factors					
Anxiety regarding ageing	9	8 (88.89)	0 (0)	1 (11.11)	+
Fear and/or salience of death	9	7 (77.78)	0 (0)	2 (28.57)	+
Conscientiousness personality	3	0 (0)	2 (66.66)	1 (33.33)	-
Agreeableness personality	3	0 (0)	3 (100)	0 (0)	-
Extraverted personality	3	0 (0)	2 (66.66)	1 (33.33)	-
Level of personal collectivism	3	0 (0)	2 (66.66)	1 (33.33)	-
**Interpersonal and intergroup level**					
Frequency of contact with older people in general	29	0 (0)	9 (31.03)	20 (68.97)	ns/mix
Target’s age (older)	27	17 (62.96)	2 (7.40)	8 (29.62)	-
Target’s sex (being a woman)	21	9 (42.85)	3 (14.29)	9 (42.85)	ns/mix
Frequency of contact with grandparents and other relatives	18	1 (5.56)	10 (55.55)	7(38.89)	ns/mix
Quality of contact with older people in general	13	0 (0)	10 (76.92)	3 (23.07)	-
Older people presented negatively	14	13 (92.85)	0 (0)	1 (7.69)	+
Older people presented positively	13	0 (0)	13 (100)	0 (0)	-
Quality of contact with grandparents and other relatives	10	0 (0)	7 (70)	3 (30)	-
Voluntary and paid experience with older people	8	0 (0)	4 (50)	4 (50)	ns/mix
**Institutional and cultural level**					
Available economic resources	5	0 (0)	3 (60)	2 (40)	-
Percentage of older people in the country	3	2 (66.66)	0 (0)	1 (33.33)	+

Note: Pos—Positive association with ageism (i.e., the determinant is associated with higher levels of ageism); Neg—Negative association with ageism (i.e., the determinant is associated with lower levels of ageism); ns/mix—no-significant or mixed findings in the relation between the determinant and ageism levels; Assoc—Association; + positive association with ageism; - negative association with ageism; n = number of papers.

**Table 3:** Distribution of the robust determinants of “other-directed” ageism by the age of the participants.

Determinants (n = number of Overall Papers)	Y (n)	Direction of the Association with Ageism (n, %)			Assoc (+, −, ns /mix)	O (n)	Direction of the Association with Ageism (n, %)			Assoc (+, −, ns /mix)
		Pos	Neg	ns /mix			Pos	Neg	ns /mix	
Intrapersonal level										
Behavioural and psychosocial factors										
Anxiety regarding ageing (n = 9)	5	4 (80)	0 (0)	1 (20)	+	3	3 (10)	0 (0)	0 (0)	+
Fear and/or salience of death (n = 9)	5	4 (80)	0 (0)	1 (20)	+	4	4 (10)	0 (0)	0 (0)	+
Conscientiousness personality (n = 3)	3	0 (0)	2 (66.66)	1 (33.33)	-	2	0 (0)	1 (50)	1 (50)	*
Agreeableness personality (n = 3)	3	0 (0)	3 (100)	0 (0)	-	2	0 (0)	2 (100)	0 (0)	*
Extraverted personality (n = 3)	3	0 (0)	2 (66.67)	1 (33.33)	-	2	0 (0)	2 (100)	0 (0)	*
Level of personal collectivism (n = 3)	3	0 (0)	2 (66.67)	1 (33.33)	-	1	0 (0)	1 (100)	0 (0)	*
Interpersonal and intergroup level										
Target’s age (older) (n = 27)	25	15 (60)	2 (8)	8 (32)	+	10	7 (70)	1 (10)	2 (20)	+
Quality of contact with older people in general (n = 13)	13	0 (0)	10 (76.92)	3 (23.08)	-	1	0 (0)	1 (100)	0 (0)	*
Older people presented negatively (n = 14)	14	13 (92.86)	0 (0)	1 (7.14)	+	3	3 (10)	0 (0)	0 (0)	+
Older people presented positively (n = 13)	13	0 (0)	13 (100)	0 (0)	-	3	0 (0)	3 (100)	0 (0)	-
Quality of contact with grandparents and other relatives (n = 10)	10	0 (0)	7 (70)	3 (30)	-	1	0 (0)	1 (100)	0 (0)	*
Institutional and cultural level										
Available economic resources (n = 5)	5	0 (0)	3 (60)	2 (40)	-	3	0 (0)	2 (66.6)	1 (33.3)	-
Percentage of older people in the country (n = 3)	2	1 (50)	0	1 (50)	*	1	0 (0)	0 (0)	1 (100)	*

Note: Pos—Positive association with ageism (i.e., the determinant is associated with higher levels of ageism); Neg—Negative association with ageism (i.e., the determinant is associated with lower levels of ageism); ns/mix—no-significant or mixed findings in the relation between the determinant and ageism levels; Assoc—Association; + positive association with ageism; - negative association with ageism; n = number of papers; Y (n) = number of paper including younger participants; O (n) = number of papers including older participants; * cases that do not involve tree or more papers exploring the determinants for that age group and hence are not considered for further analyses.

**Table 4:** Determinants of “self-directed forms of ageism” (total N = 20).

Determinants	Number Overall Papers (n ≥ 3)	Direction of the Association with Ageism (n, %)			Association (+,−,ns/mix)
		Pos	Neg	ns/mix	
**Intrapersonal Level**					
**Demographics (participants)**					
Age (older)	14	2 (15.38)	7 (53.85)	5 (38.46)	ns/mix
Sex (being a male)	9	4 (44.44)	1 (11.11)	4 (44.44)	ns/mix
Better physical and mental health condition	9	0 (0)	8 (88.89)	1 (11.11)	-
Years of education	6	2 (33.33)	2 (33.33)	2 (33.33)	ns/mix
Marital status (being married)	5	0 (0)	1 (20)	4 (80)	ns/mix
Ethnicity: Black vs. White	4	1 (25)	1 (25)	2 (50)	ns/mix
Ethnicity: Lat/Hisp vs. White	4	1 (25)	1 (25)	2 (50)	ns/mix
Socio-economic status	4	0 (0)	2 (50)	2 (50)	ns/mix
Employment status	3	0 (0)	0 (0)	3 (100)	ns/mix

Note: Pos—Positive association with ageism (i.e., the determinant is associated with higher ageism levels); Neg—Negative association with ageism (i.e., the determinant is associated with lower levels of ageism); ns/mix—no-significant or mixed findings in the relation between the determinant and ageism levels; Assoc—Association; + positive association; - negative association.

## Supplementary Materials

The following are available online at https://www.mdpi.com/1660-4601/17/7/2560/s1, Table S1: Inclusion and exclusion criteria for study selection; Table S2: Search string for Pubmed search; Table S3: Country of origin and measure of ageism of studies included in this review; Table S4: Determinants of “other-directed” forms of ageism explored in more than three studies (total N = 188); Table S5: Determinants of “other-directed” forms of ageism explored in less than three studies (total N = 188); Table S6: Determinants of “self-directed” forms of ageism explored in more than three studies (total N = 20); Table S7: Determinants of “self-directed” forms of ageism explored in less than three studies (total N = 20); Figure S1: Summary of the quality of the studies assessment; Table S8. Quality of the evidence score; Table S9: PRISMA checklist.
