# Supplementary material for: Determinants of Ageism against Older Adults: A Systematic Review
Source: Int J Environ Res Public Health. 2020 Apr 8;17(7):2560. doi: 10.3390/ijerph17072560 (PMC7178234; doi:10.3390/ijerph17072560)
Supplement: Supplementary file 1 [file ijerph-17-02560-s001.pdf]

# – Supplementary Materials –

Marques, S., Mariano, J., Mendonça, J., De Tavernier, W., Hess, M., Naegele, L., Peixeiro, F. & Martins, D. (2020). Determinants of ageism against older adults: a systematic review

Table S1. Inclusion and exclusion criteria for study selection

**Inclusion criteria**	
	Studies focusing on ageism towards older adults (defined in this study as studies including targets aged 50 years or older to include studies conducted also with older workers)
	Studies aiming to explore determinants of ageism
	Studies using an ageism measure as the dependent variable
	Quantitative studies
	Studies published after 1969
	Full text available in English, French, or Spanish
**Exclusion criteria**	
	Studies focusing on ageism towards younger age groups (i.e., target of ageism aged under 50 years)
	Studies using ageism measures as both independent and dependent variables
	Qualitative and mixed methods studies
	Literature reviews and meta-analysis
	Non-empirical articles (e.g., theoretical discussions, opinion pieces, book reviews)
	Studies only describing prevalence
	Studies evaluating interventions
	Studies with exclusive methodological aims (e.g., measurement validation)
	Studies published before 1970
	Articles written in languages other than English, French, or Spanish
	Full text unavailable or inexistent (e.g., conference abstracts)

Table S2. Search String for Pubmed search

Concept	Search string
**Ageism**	(“Ageism”[MeSH] OR ageism[TiAb] OR agism[TiAb] OR ageist[TiAb] OR agist[TiAb] OR “age discrimination”[TiAb] OR “age prejudice”[TiAb] OR “age stereotype”[TiAb] OR “self perceptions of ageing”[TiAB] OR “self perceptions of aging”[TiAB] OR "age identity"[Tiab])<br>OR<br>(("Aged"[Mesh] OR “Aged, 80 and over”[MeSH] OR “Frail Elderly”[MeSH] OR elder*[TiAb] OR “seniors”[TiAb] OR “older adult”[TiAb] OR “older person”[TiAb] OR “older adults”[TiAb] OR “older persons”[TiAb] OR “older peoples”[tiab] OR “older people”[TiAb] OR “aging”[MeSH] OR “ageing”[TiAb] OR “aging”[TiAb] OR “Old age”[Ti]) AND ("Social Exclusion"[Tiab] OR "social rejection"[TIab] OR "Social Acceptance"[TIab] OR “stereotyped behavior”[Mesh] OR “social perception”[Ti] OR “age identification”[ti] OR “self-perceptions”[tiAb] OR “Prejudice”[MeSH] OR “prejudice”[TiAb] OR stereotyp*[TiAb] OR “Stereotyping”[MeSH] OR "Social Discrimination"[Mesh] OR “Intergenerational Relations”[Mesh]))<br>NOT (“Animals” NOT ("Animals"[Mesh] AND "Humans"[Mesh]))<br>NOT Plants[Mesh]

Table S3. Country of origin and measure of ageism of studies included in this review

First author (date)	Country	Measure
Adams-Price [36]	US	Other
Allan et al. (2014)[37]	Canada	FSA
Ayalon (2013)[38]	Multiple countries	Other
Ayalon (2016)[39]	US	PAD
Bacanli et al. (1994)[40]	Turkey Finland	Other
Baker (1983)[41]	Canada	Other
Beatty (2009)[42]	US	ASD
Beck et al. (1979)[43]	US	ASD
Bell & Stanfield (1973)[44]	US	ASD
Bergman et al. (2013)[45]	Israel	FSA, PAM
Bhana (1983)[46]	India	ACL
Bieman-Copland & Ryan (2001)[47]	Canada	Other
Bierly (1985)[48]	US	KOPS, TLQ
Bodner & Cohen-Fridel (2014)[49]	Israel	FSA
Bodner & Cohen-Friedel (2010)[50]	Israel	FSA
Bodner & Lazar (2008)[51]	Israel	FSA
Bodner et al. (2011)[52]	Israel	FSA
Bodner et al. (2012)[53]	Israel	FSA
Bodner et al. (2015)[54]	Israel	FSA
Boudjemadi & Gana (2012)[55]	France	IAT
Bousfield & Hutchison (2010)[56]	UK	Other
Bowen & Skirbekk (2013)[57]	Multiple countries	Other
Braithwaite et al. (1986)[58]	Australia	ASD

First author (date)	Country	Measure
Braithwaite et al. (1993)[59]	Australia	Other
Brewer & Lui (1984)[60]	US	Other
Bryant et al. (2014)[61]	Australia	AAQ
Burge (1978)[62]	US	TLQ
Canetto et al. (1995)[63]	US	Other
Cary et al. (2013)[64]	Canada	Other
Caspi (1984)[65]	UK	Other
Celejewski & Dion (1998)[66]	Canada	ASD
Chan et al. (2012)[67]	Multiple countries	Other
Chang et al. (1984)[68]	US Taiwan	KOPS, NOP
Chasteen (2000)[69]	Canada	SVES, Other
Chasteen (2005)[70]	Canada	Other
Chen et al. (2010)[71]	US	Other
Chen et al. (2017)[72]	US	Other
Cherry et al. (2015)[73]	US	FSA, ROPE
Cheung et al. (1999)[74]	Hong Kong	Other
Cheung et al. (2011)[75]	China	Other
Chiu et al. (2001)[76]	UK Hong Kong	Other
Choi et al. (2013)[77]	Mongolia US	Other
Chonody & Teater (2016)[15]	US UK (England) Australia	ROPE, Other
Chopik & Giasson (2017)[78]	US	IAT, Other
Chou & Choi (2011)[79]	US	Other
Chung & Lin (2012)[80]	US China	VAT

First author (date)	Country	Measure
Clément-Guillotin et al. (2015)[81]	France	Other
Collette-Pratt (1976)[82]	US	Other
Connor et al. (1978)[83]	US	Other
Cox & Barron (2012)[84]	US	Other
Crew (1984)[85]	US	Other
Cullen et al. (2009)[86]	Ireland	Other
DaŞBaŞ & Kesen (2015)[87]	Turkey	AAS
Dasgupta & Greenwald (2001)[88]	US	IAT, Other
Davidson et al. (2008)[89]	US China	Other
De Guzman et al. (2014)[90]	Philippines	Other
Demir et al. (2016)[91]	Turkey	ASS
Depaola et al. (1992)[92]	US	SVES
Depaola et al. (1994)[93]	US	SVES
Depaola et al. (2003)[94]	US	SVES, SADS
de Paula Couto & Koller (2012)[95]	Brazil	Other
Deuisch et al. (1986)[96]	US	Other
Diekman & Hirnisey (2007)[97]	US	Other
Donlon et al. (2005)[98]	US	IAM
Drury et al. (2016)[99]	UK	Other
Drydakis et al. (2018)[100]	UK	Other
Duncan & Schaller (2009)[101]	Canada	IAT
Faulkner et al. (2007)[102]	Canada	Other
Ferraro (1992)[103]	US	Other
Finkelstein & Burke (1998)[104]	US	Other

First author (date)	Country	Measure
Folwell (1997)[105]	US	AGED, LIA
Freeman (2002)[106]	US	AGED
Fullen (2016)[107]	US	IAS
Fusilier & Hitt (1983)[108]	US	Other
Gattuso & Saw (1998)[109]	Australia	RAQ
Gattuso & Shadbolt (2002)[110]	Australia Fiji/Pacific Islands	RAQ
Gekoski & Knox (1990)[111]	Canada	ASD
Gekoski et al. (1984)[112]	Canada	ASD
Gibson et al. (1993)[113]	Canada	Other
Gluth et al. (2010)[114]	Germany	ASD
Gordon et al. (1988)[115]	US	Other
Graham & Baker (1989)[116]	Canada	Other
Hale (1998)[117]	US	Other
Harris & Fiedler (1988)[118]	US	TLQ
Harwood et al. (1994)[119]	US Hong Kong	SVQ
Harwood et al. (2001)[120]	Australia China Hong Kong Philippines Thailand	Other
Harwood et al. (2005)[121]	US UK	Other
Haught et al. (1999)[122]	US	FAQ
Hawkins (1996)[123]	US	Other
Hehman et al. (2012)[124]	US	Other
Hertzman & Zhong (2016)[125]	US	Other
Huang (2013)[126]	Multiple countries	ASD, FAQ1

First author (date)	Country	Measure
Hughes et al. (2016)[127]	US	FSA, ROPE
Hummert et al. (1997)[128]	US	Other
Hummert et al. (2002)[129]	US	IAT, Other
Hummert (1993)[130]	US	Other
Hummert (1994)[131]	US	Other
Iweins et al. (2012)[132]	Belgium	Other
Jackson & Sullivan (1988)[133]	US	KOPS, Other
Janečková et al. (2013)[134]	Czech Republic	AAQ
John (2013)[135]	Hungary	
Kalavar (2001)[136]	US	FSA, Other
Kane (2006)[137]	US	PEC
Karpinska et al. (2011)[138]	Netherlands	Other
Katz (1990)[139]	US	AOS
Kirk (2015)[140]	US	FSA-R
Knox & Gekoski (1989)[141]	US	ASD
Knox et al. (1986)[142]	Canada	FAQ, ASD
Kornadt & Kandler (2017)[143]	US	Other
Kornadt & Rothermund (2011)[144]	Germany	Other
Kornadt et al. (2013)[145]	Germany	Other
Krendl (2016)[146]	US China	KOPS, Other
Kuhlmann et al. (2017)[147]	Germany	Other
Kulik et al. (2000)[148]	US	Other
Kwong See & Nicoladis (2009)[149]	US	Other
Laditka et al. (2011)[150]	US	ASD, Other

First author (date)	Country	Measure
Laidlaw et al. (2010)[151]	UK (Scotland) China (Beijing)	AAQ
Lamont et al. (2017)[152]	UK	ATOA
Levy (1999)[153]	US Japan China	FAQ, Other
Levy (2008)[154]	US	ATOA
Levy et al. (2015)[155]	US	Other
Lin & Bryant (2009)[156]	Australia Malaysia Singapore China	FSA
Linville (1982)[157]	US	Other
Locke-Connor & Walsh (1980)[158]	US	Other
Löckenhoff et al. (2009)[159]	Multiple countries	Other
Lookinland & Anson (1995)[160]	US	KOPS
Luchesi et al. (2016)[161]	Brazil	Other
Luo et al. (2013)[162]	US China	FSA
Luszcz & Fitzgerald (1986)[163]	Australia	FAQ-R, ASD
Lytle (2016)[164]	US	FSA, IAS, FAQ, Other
Marquet et al. (2016)[165]	Belgium Burundi	FSA-R, Other
Martens et al. (2004)[166]	US	Other
McCann & Keaton (2013)[167]	US Thailand	Other
McNamara et al. (2016)[168]	US	Other
Melanson & Downe-Wamboldt (1985)[169]	US	Other
Miller et al. (1984)[170]	US	Other
Milligan et al. (1985)[171]	US	ASD

First author (date)	Country	Measure
Milligan et al. (1989)[172]	US	ASD
Montepare & Zebrowitz-McArthur (1988)[173]	US	Other
Narayan (2008)[174]	US	ASD, FAQ2
Ng et al. (2015)[175]	US	Other
Nochajski et al. (2011)[176]	US	ASD
Nochajski et al. (2009)[177]	US	ASD
North & Fiske (2013)[178]	US	Other
North & Fiske (2016)[179]	US	SIC, Other
O'Connell & Rotter (1979)[180]	US	ASD
O'Connor & McFadden (2012)[181]	US	Other
Obhi & Woodhead (2016)[182]	US	ASD, Other
Okoye (2005)[183]	US Nigeria	CAFAQ
Oliveira et al. (2015)[184]	Brazil	Other
Özdemir & Bilgili (2016)[185]	Turkey	Other
Paris et al. (1997)[186]	US	ASD
Passuth & Cook (1985)[187]	US	Other
Pecchioni & Croghan (2002)[188]	US	Other
Randler et al. (2014)[189]	Germany	Other
Reed et al. (1992)[190]	US	FAQ, KOPS
Revenson (1989)[191]	US	ASD
Rittenour & Cohen (2016)[192]	US	Other
Roberts (2008)[193]	US	FAQ
Robertson & Weiss (2017)[194]	Multiple countries	SSS, Other
Ruiz et al. (2015)[195]	US	IAT, FSA, Other

First author (date)	Country	Measure
Runkawatt et al. (2013)[196]	Thailand Sweden	KOPS
Ruscher & Hurley (2000)[197]	US	Other
Ryan et al. (2004)[198]	Canada Hong Kong China Taiwan South Korea	LIA
Ryan & Laurie (1990)[199]	Canada	Other
Sanders & Pittman (1987)[200]	US	Other
Sargent-Cox et al. (2012)[201]	Australia	ATOA
Sheier et al. (1978)[202]	US	Other
Schwartz & Simmons (2001)[203]	US	Other
Sherman et al. (1978)[204]	US	Other
Sherman et al. (1985)[205]	US	Other
Signori et al. (1982)[206]	Canada	Other
Skorinko & Sinclair (2013)[207]	US	Other
Smith et al. (2017)[208]	US	KOPS
Soliz & Harwood (2003)[209]	US	Other
Solomon & Vickers (1979)[210]	US	TLQ
Springer & Harwood (2015)[211]	US	Other
Steitz & Verner (1987)[212]	US	FAQ
Stewart et al. (2005)[213]	New Zealand	FAQ1, KOPS
Stewart & Ryan (1982)[214]	US	Other
Stier & Kline (1980)[215]	US	FAQ, ASD
Stokes & Moorman (2016)[216]	US	PD
Tam et al. (2006)[217]	UK	IAT, Other

First author (date)	Country	Measure
Tan et al. (2004)[218]	China	Other
Thorson et al. (1974)[219]	US	KOPS
Tomko & Munley (2013)[220]	US	ASD,<br>Other
Trigg et al. (2012)[221]	UK	AAQ
Turner & Crisp (2010)[222]	UK	IAT,<br>Other
Vauclair et al. (2015)[223]	Multiple<br>countries	Other
Vauclair et al. (2017)[1] [224]	UK<br>Taiwan	Other
Vauclair et al. (2017)[2][225]	Portugal	Other
Verhaeghen et al. (2011)[226]	US	AGED
Vrugt & Schabracq (1996)[227]	Netherlands	Other
Waldrop & Gress (2003)[228]	US	FAQ2,<br>Other
Wang et al. (2009)[229]	Taiwan	KOP
Wingard et al. (1982)[230]	US	TLQ (ad.)
Wurm et al. (2014)[231]	Germany	ATOA
Zhang et al. (2016)[232]	China<br>WVS	SIC<br>Other
Zweibel et al. (1993)[233]	US	Other

Note. FSA: Fraboni Aging Scale; PAD: Perceived Age Discrimination; KOPS: Kogan´s Old People Scale; ASD: Aging Semantic Differential; PAM: Perceptions of Aging Measure; ACL: Adjective Check List; TLQ: Tuckman Lorge-Questionnaire; IAT: Implicit Association Test; NOP: Needs of Older People; SVES: Social Value of the Elderly Scale; ROPE: Relating to Older People Evaluation; VAT: Views on Aging Task; AAS: Ageism Attitude Scale; SADS: Stereotypic Age Decrement Scale; IAM: Image of Aging Measure; AGED: Age Group Evaluation and Description Inventory; Lia: Language in Adulthood Scale; AAQ: Attitudes to Aging Questionnaire ; FAQ: Palmore Facts on Aging Quiz; SSS: MacArthur Scale of Subjective Social Status; RAQ: Reactions to Ageing Questionnaire; PEC: Perceptions of elder capacity; CAFAQ: Child-adolescent facts on Aging Quiz; SIC: Succession, Identity, and Consumption Ageism Scale; ATOA: Attitude toward own aging; Other: other measures of ageism besides the already identified

Table S4. Determinants of "other-directed" forms of ageism explored in more than three studies (total *N* = 188)

Variables ($n \geq 3$)	% overall studies	Direction of the association (n)	Reference number	n/N (%)	(+, -, NS/Mix)
			Intrapersonal level		
Demographics (participants)					
Age (older) (n = 81)	43.08	Pos: 8	[94,128,161,172,189,219,223,225]	9.88	NS/Mix
		Neg: 32	[41,59,60,66,76,92,93,95,103,113,116,122, 130,132,133,136,139,140,144,145,153,163, 168,178,185-187,190,198,206,213,220]	39.50	
		NS/Mix: 41	[42-44,50-53,62,63,67,70,71,73,74,78,81,82,91,98,99, 104,108,114,115,117,123,129,135,143,164, 167,169,177,184,191,194,196,215,224,228, 233]	50.62	
Sex (being a male) (n = 67)	35.64	Pos: 23	[15,37,48,53,55,59,68,76,78,82,83,87,122, 123,136,139,160,162,164,166,192,208,229]	34.32	NS/Mix
		Neg: 3	[177,220,223]	4.47	
		NS/Mix: 41	[36,40-42,45,46,50,52,62,77,81,91,94,99,101-103,108,111,112,118,128,132,137,145,159, 161,168,183,184,186,187,189,195,206,210, 212,213,218,230]	61.19	
Years of education (n = 24)	12.77	Pos: 2	[161,168]	8.33	NS/Mix
		Neg: 7	[78,87,103,137,139,205,219]	29.17	
		NS/Mix: 15	[37,38,45,50,91,98,132,140,160,164,167, 169,184,187,218]	62.50	
Cultural background: East vs West (n = 18)	9.57	Pos: 4	[101,119,126,162]	22.22	NS/Mix
		Neg: 1	[80]	5.56	
		NS/Mix: 13	[68,76,77,89,120,146,153,156,159,167,196, 198,224]	72.22	
Ethnicity (n = 13)					
Black vs White (n = 13)	6.91	Pos: 5	[62,85,94,118,208]	38.46	NS/Mix
		Neg: 0		0	
		NS/Mix: 8	[37,42,108,124,164,184,187,211]	61.53	

Variables (*n* ≥ 3)	% overall studies	Direction of the association (n)	Reference number	n/N (%)	(+, -, NS/Mix)
Latino/Hispanic vs White (n = 7)	3.72	Pos: 2	[118,122]	28.57	NS/Mix
		Neg: 0		0	
		NS/Mix: 5	[37,42,124,164,211]	71.42	
Asian vs White (n = 6)	3.19	Pos: 0		0	NS/Mix
		Neg: 0		0	
		NS/Mix: 6	[37,42,124,164,195,211]	100	
Study area related with ageing & care (n = 7)	3.72	Pos: 1	[87]	14.28	NS/Mix
		Neg: 2	[137,182]	28.57	
		NS/Mix: 4	[43,91,169,186]	57.14	
Professional experience in general (n = 6)	3.19	Pos: 0		0	NS/Mix
		Neg: 3	[62,160,220]	50	
		NS/Mix: 3	[74,168,191]	50	
Better physical and mental health condition (n = 6)	3.19	Pos: 0		0	NS/Mix
		Neg: 1	[233]	16.67	
		NS/Mix: 5	[50,52,98,140,171]	83.33	
Socio-economic status (n = 6)	3.19	Pos: 0		0	NS/Mix
		Neg: 0		0	
		NS/Mix: 6	[45,50,91,140,164,187]	100	
Degree of religiosity (n = 5)	2.66	Pos: 0		0	NS/Mix
		Neg: 2	[38,232]	40	
		NS/Mix: 3	[45,48,62]	60	

Variables ($n \geq 3$)	% overall studies	Direction of the association (n)	Reference number	n/N (%)	(+, -, NS/Mix)
Living in urban (vs rural) context (n = 5)	2.13	Pos: 2	[38,161]	40	NS/Mix
		Neg: 0		0	
		NS/Mix: 3	[91,109,169]	60	
Marital status (being married) (n = 3)	1.60	Pos: 0		0	NS/Mix
		Neg: 1	[233]	33.33	
		NS/Mix: 2	[37,45]	66.66	
Behavioural and psychosocial factors					
Anxiety regarding ageing (n = 9)	4.79	Pos: 8	[37,54,69,92-94,99,125]	88.89	+
		Neg: 0		0	
		NS/Mix: 1	[56]	11.11	
Fear and/or salience of death (n = 9)	4.79	Pos: 7	[15,49,54,82,92,94,166]	77.78	+
		Neg: 0		0	
		NS/Mix: 2	[55,93]	28.57	
Personality traits (n = 3)					
Conscientious-ness (n = 3)	1.60	Pos: 0		0	-
		Neg: 2	[37,143]	66.6	
		NS/Mix: 1	[114]	33.3	
Agreeableness (n = 3)	1.60	Pos: 0		0	-
		Neg: 3	[37,114,143]	100	
		NS/Mix: 0		0	
Extraversion (n = 3)	1.60	Pos: 0		0	-
		Neg: 2	[114,143]	66.66	
		NS/Mix: 1	[37]	33.3	

Variables (*n* ≥ 3)	% overall studies	Direction of the association (n)	Reference number	n/N (%)	(+, -, NS/Mix)
Personal collectivism (n = 3)	1.60	Pos: 0		0	-
		Neg: 2	[42,232]	66.66	
		NS/Mix: 1	[224]	33.33	
Interpersonal and intergroup level					
Frequency of contact with older people in general (n = 29)	15.43	Pos: 0	-	0	NS/Mix
		Neg: 9	[42,117,123,127,149,162,208,213,218]	31.03	
		NS/Mix: 20	[37,59,65,74,76,82,89,97,99,118,124,159, 160,169,170,176,191,203,212,217]	68.97	
Target's age (older) (n = 27)	13.83	Pos: 17	[36,46,55,63,77,86,104,113,116,123,158, 166,171,180,199,214,227]	62.96	NS/Mix
		Neg: 2	[202,204]	7.40	
		NS/Mix: 8	[41,44,58,84,112,119,181,198]	29.63	
Target's sex (being a women) (n = 21)	11.17	Pos: 9	[46,55,96,105,116,124,128,131,218]	42.85	NS/Mix
		Neg: 3	[123,150,174]	14.29	
		NS/Mix: 9	[41,58,63,66,112,124,145,158,180]	42.85	
Frequency of contact with grandparents and older family members (n = 18)	9.57	Pos: 1	[184]	5.56	NS/MIX
		Neg: 10	[68, 123, 127, 149, 162, 164, 185, 190, 213, 218]	55.55	
		NS/Mix: 7	[59, 65, 82, 89, 91, 124, 176]	38.89	
Quality of contact with older people in general (n = 13)	6.91	Pos: 0		0	-
		Neg: 10	[42,56,99,117,123,125,142,182,203,218]	76.92	
		NS/Mix: 3	[169,212,217]	23.07	
Older persons presented negatively (n = 14)	6.91	Pos: 13	[43,47,67,72,111,128,131,138,172,173,197, 202,214]	92.85	+
		Neg: 0		0	
		NS/Mix: 1	[181]	7.69	
	6.91	Pos: 0		0	-

**Variables (*n* ≥ 3)**	**% overall studies**	**Direction of the association (n)**	**Reference number**	**n/N (%)**	**(+, -, NS/Mix)**
Older persons presented positively (n = 13)		Neg: 13	[69,72,81,83,86,88,89,102,108,128,138,158,215]	100	
		NS/Mix: 0		0	
Quality of contact with grandparents and other relatives (n = 10)	5.32	Pos: 0		0	-
		Neg: 7	[87,123,127,182,188,217,218]	70	
		NS/Mix: 3	[106,121,169]	30	
Voluntary and paid experience with older adults (n = 8)	4.26	Pos: 0		0	NS/Mix
		Neg: 4	[134, 176, 182, 185]	50	
		NS/Mix: 4	[123, 161, 208, 218]	50	
			Institutional and environmental level		
Available economic resources (n = 5)	2.66	Pos: 0		0	-
		Neg: 3	[165,179,223]	60	
		NS/Mix: 2	[38,159]	40	
Percentage of older people in the country (n = 3)	1.60	Pos: 2	[159,175]	66.66	+
		Neg: 0		0	
		NS/Mix: 1	[38]	33.33	

Note: Pos – Positive association with ageism (i.e., the determinant is associated with higher ageism levels); Neg – Negative association with ageism (i.e., the determinant is associated with lower levels of ageism); NS/Mix – non-significant or mixed findings in the relation between the determinant and ageism levels; Assoc – Association; + positive association; - negative association

Table S5. Determinants of "other-directed" forms of ageism explored in less than three studies (total *N* = 188)

Variables (n ≤ 2)	% overall studies	Direction of the association (n)	Reference number	n/N (%)	(+, -, NS/Mix)
		Intrapersonal level			
Demographics (participants)					
Cultural background: other (n = 2)	1.06	Pos: 0		0	NS/Mix
		Neg: 0		0	
		NS/Mix: 2	[40,110]	100	
Size of hometown (n = 2)	1.06	Pos: 0		0	NS/Mix
		Neg: 0		0	
		NS/Mix: 2	[123,218]	100	
Political orientation (n = 2)	1.06	Pos: 0		0	NS/Mix
		Neg: 0		0	
		NS/Mix: 2	[48,211]	100	
Multicultural experience (n = 1)	0.53	Pos: 0		0	-
		Neg: 1	[220]	100	
		NS/Mix: 0		0	
Experience of stressful events (n = 1)	0.53	Pos: 0		0	NS/Mix
		Neg: 0		0	
		NS/Mix: 1	[155]	100	
Being an only child (n = 1)	1.06	Pos: 0		0	NS/Mix
		Neg: 0		0	
		NS/Mix: 1	[218]	50	
Behavioural and psychosocial factors					

**Variables (n ≤ 2)**	**% overall studies**	**Direction of the association (n)**	**Reference number**	**n/N (%)**	**(+, -, NS/Mix)**
Empathy (n = 2)	1.06	Pos: 0		0	NS/Mix
		Neg: 1	[49]	50	
		NS/Mix: 1	[37]	50	
Attachment style (n = 2)					
Insecure/anxious attachment (n = 2)	1.06	Pos: 1	[49]	50	NS/Mix
		Neg: 0		0	
		NS/Mix: 1	[50]	50	
Avoidant attachment (n = 2)	1.06	Pos: 0		0	NS/Mix
		Neg: 0		0	
		NS/Mix: 2	[49,50]	100	
Identification with the young age group (n = 2)	1.06	Pos: 2	[64,70]	100	+
		Neg: 0		0	
		NS/Mix: 0		0	
Openness (n = 2)	1.06	Pos: 0		0	-
		Neg: 2	[37,143]	100	
		NS/Mix: 0		0	
Neuroticism (n = 2)	1.06	Pos: 1	[143]	50	NS/Mix
		Neg: 0		0	
		NS/Mix: 1	[37]	50	
Life satisfaction (n = 2)	1.06	Pos: 0		0	NS/Mix
		Neg: 0		0	
		NS/Mix: 2	[161,187]	100	
Cognitive overload (n = 1)	0.53	Pos: 1	[148]	100	+

Variables (n ≤ 2)	% overall studies	Direction of the association (n)	Reference number	n/N (%)	(+, -, NS/Mix)
		Neg: 0		0	
		NS/Mix: 0		0	
Ethics knowledge (n = 1)	0.53	Pos: 0		0	NS/Mix
		Neg: 0		0	
		NS/Mix: 1	[193]	100	
Rigidity personality (n = 1)	0.53	Pos: 1	[154]	100	+
		Neg: 0		0	
		NS/Mix: 0		0	
Grateful disposition (n = 1)	0.53	Pos: 0		0	-
		Neg: 1	[37]	100	
		NS/Mix: 0		0	
Acceptance of view of natural way of life (n = 1)	0.53	Pos: 1	[233]	100	+
		Neg: 0		0	
		NS/Mix: 0		0	
Social desirability (n = 1)	0.53	Pos: 0		0	NS/Mix
		Neg: 0		0	
		NS/Mix: 1	[73]	100	
Self-esteem (n = 1)	0.53	Pos: 0		0	NS/Mix
		Neg: 0		0	
		NS/Mix: 1	[74]	100	
Sexism (n = 1)	0.53	Pos: 0		0	NS/Mix

Variables (n ≤ 2)	% overall studies	Direction of the association (n)	Reference number	n/N (%)	(+, -, NS/Mix)
		Neg: 0		0	
		NS/Mix: 1	[15]	100	
Positive attitudes toward health, achievement and productivity (n = 1)	0.53	Pos: 1	[82]	100	+
		Neg: 0		0	
		NS/Mix: 0		0	
Perceived power distance between young and old (n = 1)	0.53	Pos: 1	[84]	100	+
		Neg: 0		0	
		NS/Mix: 0		0	
Willingness to live and work with older people in the future (n = 2)	0.53	Pos: 0		0	-
		Neg: 1	[91]	100	
		NS/Mix: 1	[218]	0	
Perspective-taking (n = 2)	1.06	Pos: 0		0	NS/Mix
		Neg: 1	[121]	50	
		NS/Mix: 1	[207]	50	
Self-disclosure (n = 2)	0.53	Pos: 0		0	-
		Neg: 2	[99,121]	100	
		NS/Mix: 0		0	
Ingroup norms (n = 1)	0.53	Pos: 0		0	-
		Neg: 1	[99]	100	
		NS/Mix: 0		0	
Optimism (n = 1)	0.53	Pos: 0		0	-

**Variables (n ≤ 2)**	**% overall studies**	**Direction of the association (n)**	**Reference number**	**n/N (%)**	**(+, -, NS/Mix)**
		Neg: 1	[168]	100	
		NS/Mix: 0		0	
Low anxiety trait (n = 1)	0.53	Pos: 0		0	-
		Neg: 1	[139]	100	
		NS/Mix: 0		0	
Sensitive-intuitive trait (n = 1)	0.53	Pos: 0		0	-
		Neg: 1	[139]	100	
		NS/Mix: 0		0	
Higher intellectual ability trait (n = 1)	0.53	Pos: 0		0	-
		Neg: 1	[139]	100	
		NS/Mix: 0		0	
Low anxiety personality traits (n = 1)	0.53	Pos: 0		0	-
		Neg: 1	[139]	100	
		NS/Mix: 0		0	
More complex representation of older people (n = 1)	0.53	Pos: 0		0	-
		Neg: 1	[157]	100	
		NS/Mix: 0		0	
Confrontation with ageing and the ageing self (n = 1)	0.53	Pos: 1	[192]	100	+
		Neg: 0		0	
		NS/Mix: 0		0	
More perceived stress	0.53	Pos: 1	[161]	100	+

Variables (n ≤ 2)	% overall studies	Direction of the association (n)	Reference number	n/N (%)	(+, -, NS/Mix)
(n = 1)		Neg: 0		0	
		NS/Mix: 0		0	
Individuation of grandparents (n = 1)	0.53	Pos: 0		0	NS/Mix
		Neg: 0		0	
		NS/Mix: 1	[121]	100	
Perceived vulnerability to disease (n = 1)	0.53	Pos: 0		0	NS/Mix
		Neg: 0		0	
		NS/Mix: 1	[101]	100	
Positive affect (n = 1)	0.53	Pos: 0		0	-
		Neg: 1	[114]	100	
		NS/Mix: 0		0	
Negative affect (n = 1)	0.53	Pos: 1	[114]	100	+
		Neg: 0		0	
		NS/Mix: 0		0	
Identity assimilation (n = 1)	0.53	Pos: 1	[140]	100	+
		Neg: 0		0	
		NS/Mix: 0		0	
Identity accommodation (n = 1)	0.53	Pos: 1	[140]	100	+
		Neg: 0		0	
		NS/Mix: 0		0	
Identity balance (n = 1)	0.53	Pos: 0		0	-

Variables (n ≤ 2)	% overall studies	Direction of the association (n)	Reference number	n/N (%)	(+, -, NS/Mix)
		Neg: 1	[140]	100	
		NS/Mix: 0		0	
		Interpersonal and intergroup level			
Target-self similarity (n = 2)	1.06	Pos: 0		0	-
		Neg: 2	[60,150]	100	
		NS/Mix: 0		0	
Extended contact with older people (n = 2)	0.53	Pos: 0		0	-
		Neg: 2	[99,164]	100	
		NS/Mix: 0		0	
Target ethnicity (being black) (n = 1)	0.53	Pos: 1	[100]	100	+
		Neg: 0		0	
		NS/Mix: 0		0	
Known vs general older target (n = 1)	0.53	Pos: 0		0	-
		Neg: 1	[200]	100	
		NS/Mix: 0		0	
Variations in perceptions of grandparents relationship (n = 1)	0.53	Pos: 0		0	NS/Mix
		Neg: 0		0	
		NS/Mix: 1	[209]	100	
Accommodation in grandparents relationship (n = 1)	0.53	Pos: 0		0	-
		Neg: 1	[121]	100	
		NS/Mix: 0		0	
	0.53	Pos: 0	[121]	100	+

Variables (n ≤ 2)	% overall studies	Direction of the association (n)	Reference number	n/N (%)	(+, -, NS/Mix)
Anxiety in grandparents relationship (n = 1)		Neg: 1		0	
		NS/Mix: 0		0	
Variations in perceptions of grandparents relationship (n = 1)	0.53	Pos: 0		0	NS/Mix
		Neg: 0		0	
		NS/Mix: 1	[209]	100	
Imagined contact with older people (n = 1)	0.53	Pos: 0		0	-
		Neg: 1	[222]	100	
		NS/Mix: 0		0	
Institutional and environmental level					
Percentage of older people in paid or voluntary work (n = 2)	1.06	Pos: 0		0	-
		Neg: 2	[57,223]	100	
		NS/Mix: 0		0	
Level of education in the country (n = 2)	1.06	Pos: 0		0	NS/Mix
		Neg: 1	[223]	50	
		NS/Mix: 1	[38]	50	
Presence of an anti-age discrimination policy (n = 2)	1.06	Pos: 0		0	-
		Neg: 2	[76,84]	100	
		NS/Mix: 0		0	
Media exposure (n = 2)	1.06	Pos: 1	[98]	50	NS/Mix
		Neg: 0		0	
		NS/Mix: 1	[187]	50	
Accountability/ self-awareness	1.06	Pos: 1	[115]	50	NS/Mix

Variables (n ≤ 2)	% overall studies	Direction of the association (n)	Reference number	n/N (%)	(+, -, NS/Mix)
(e.g., public vs private context) (n = 2)		Neg: 1	[71]	50	
		NS/Mix: 0		0	
Collectivistic and traditional cultures (n = 2)	1.06	Pos: 0		0	NS/Mix
		Neg: 1	[38]	50	
		NS/Mix: 1	[232]	50	
Life domain being considered (e.g., family and spirituality vs health and fitness) (n = 2)	1.06	Pos: 0		0	-
		Neg: 2	[144,145]	100	
		NS/Mix: 0		0	
Country level of modernization (n = 1)	0.53	Pos: 0		0	-
		Neg: 1	[223]	100	
		NS/Mix: 0		0	
Country values of survival (vs self-expression) (n = 1)	0.53	Pos: 0		0	NS/Mix
		Neg: 0		0	
		NS/Mix: 1	[38]	100	
More recent time of measurement (n = 1)	0.53	Pos: 0		0	-
		Neg: 1	[175]	100	
		NS/Mix: 0		0	
Comparative vs non-comparative context (n = 2)	0.53	Pos: 1	[230]	50	NS/Mix
		Neg: 0		0	
		NS/Mix: 1	[141]	50	
Noisy vs silence context	0.53	Pos: 1	[199]	100	+

Variables (n ≤ 2)	% overall studies	Direction of the association (n)	Reference number	n/N (%)	(+, -, NS/Mix)
(n = 1)		Neg: 0		0	
		NS/Mix: 0		0	
Episodic vs thematic frames (n = 1)	0.53	Pos: 1	[211]	100	+
		Neg: 0		0	
		NS/Mix: 0		0	
Associative strength of negative attributes and ageing (n = 1)	0.53	Pos: 1	[226]	100	+
		Neg: 0		0	
		NS/Mix: 0		0	
Profit oriented company (n = 1)	0.53	Pos: 1	[75]	100	+
		Neg: 0		0	
		NS/Mix: 0		0	
Social responsibility oriented company (n = 1)	0.53	Pos: 0		0	-
		Neg: 1	[75]	100	
		NS/Mix: 0		0	
Customer-driven industry (n = 1)	0.53	Pos: 0		0	-
		Neg: 1	[76]	100	
		NS/Mix: 0		0	
Need of downsizing the organizational workforce (n = 1)	0.53	Pos: 0		0	NS/Mix
		Neg: 0		0	
		NS/Mix: 1	[138]	100	
	0.53	Pos: 0		0	-

Variables (n ≤ 2)	% overall studies	Direction of the association (n)	Reference number	n/N (%)	(+, -, NS/Mix)
Incidental organizational labour shortage (n = 1)		Neg: 1	[138]	100	
		NS/Mix: 0		0	
Organization described as dynamic instead of static (n = 1)	0.53	Pos: 1	[97]	100	+
		Neg: 0		0	
		NS/Mix: 0		0	
Threat to the group status (n = 1)	0.53	Pos: 1	[70]	100	NS/Mix
		Neg: 0		0	
		NS/Mix: 0		0	
Group salience in grandparent-grandchild relationship (n = 1)	0.53	Pos: 1	[121]	100	NS/Mix
		Neg: 0		0	
		NS/Mix: 0		0	

Note: Pos – Positive association with ageism (i.e., the determinant is associated with higher ageism levels); Neg – Negative association with ageism (i.e., the determinant is associated with lower levels of ageism); NS/Mix – non-significant or mixed findings in the relation between the determinant and ageism levels; Assoc – Association; + positive association; - negative association

Table S6. Determinants of "self-directed" forms of ageism explored in more than three studies (total *n* = 20)

Variables ($n \geq 3$)	% overall studies	Direction of the association (n)	Reference number	n/N (%)	(+, -, NS/Mix)
		Intrapersonal level			
Demographics (participants)					
Age (older) (n = 14)	65	Pos: 2	[39,152]	15.38	NS/Mix
		Neg: 7	[66,69,79,153,163,216,231]	53.85	
		NS/Mix: 5	[61,70,107,134,201]	38.46	
Sex (being a male) (n = 9)	50	Pos: 4	[39,79,201,231]	44.44	NS/Mix
		Neg: 1	[134]	11.11	
		NS/Mix: 4	[66,107,152,216]	44.44	
Better physical and mental health condition (n = 9)	45	Pos: 0		0	-
		Neg: 8	[39,107,134,152,171,201,216,231]	88.89	
		NS/Mix: 1	[221]	11.11	
Years of education (n = 6)	35.29	Pos: 2	[39,201]	33.33	NS/Mix
		Neg: 2	[79,152]	33.33	
		NS/Mix: 2	[107,216]	33.33	
Marital status (being married) (n = 5)	30	Pos: 0		0	NS/Mix
		Neg: 1	[201]	20	
		NS/Mix: 4	[39,61,79,107]	80	
Ethnicity (n = 4)					
Black vs White (n = 4)	20	Pos: 1	[79]	25	NS/Mix
		Neg: 1	[39]	25	
		NS/Mix: 2	[107,216]	50	

Variables (*n* ≥ 3)	% overall studies	Direction of the association (n)	Reference number	n/N (%)	(+, -, NS/Mix)
Latino/Hispanic vs White (n = 4)	20	Pos: 1	[79]	25	NS/Mix
		Neg: 1	[39]	25	
		NS/Mix: 2	[107,216]	50	
Higher socio-economic status (n = 4)	20	Pos: 0		0	NS/Mix
		Neg: 2	[79,231]	50	
		NS/Mix: 2	[61,216]	50	
Employment status (n = 3)	15	Pos: 0		0	NS/Mix
		Neg: 0		0	
		NS/Mix: 3	[39,61,79]	100	

Note: Pos – Positive association with ageism (i.e., the determinant is associated with higher ageism levels); Neg – Negative association with ageism (i.e., the determinant is associated with lower levels of ageism); NS/Mix – non-significant or mixed findings in the relation between the determinant and ageism levels; Assoc – Association; + positive association; - negative association

Table S7. Determinants of “self-directed” forms of ageism explored in less than three studies (total *n* = 20)

Variables (n ≤ 2)	% overall studies	Direction of the association (n)	Reference number	n/N (%)	(+, -, NS/Mix)
		Intrapersonal level			
Demographics (participants)					
Cultural background: East vs West (n = 2)	10	Pos: 1	[151]	50	NS/Mix
		Neg: 0		0	
		NS/Mix: 1	[153]	50	
Perceived social support (n = 2)	10	Pos: 0		0	NS/Mix
		Neg: 1	[152]	50	
		NS/Mix: 1	[79]	50	
Living in urban (vs rural) context (n = 1)	5	Pos: 0		0	-
		Neg: 1	[109]	100	
		NS/Mix: 0		0	
Study area related with ageing & care (n = 1)	5	Pos: 0		0	NS/Mix
		Neg: 0		0	
		NS/Mix: 1	[109]	100	
Having children (n = 1)	5	Pos: 0		0	-
		Neg: 1	[134]	100	
		NS/Mix: 0		0	
Marital status (n = 1)	5	Pos: 0		0	NS/Mix
		Neg: 0		0	
		NS/Mix: 1	[61]	100	

Variables (n ≤ 2)	% overall studies	Direction of the association (n)	Reference number	n/N (%)	(+, -, NS/Mix)
Behavioural and psychosocial factors					
Rigidity personality (n = 1)	5	Pos: 1	[154]	100	+
		Neg: 0		0	
		NS/Mix: 0		0	
Openness to experience (n = 1)	5	Pos: 0		0	NS/Mix
		Neg: 0		0	
		NS/Mix: 1	[61]	100	
Conscientiousness (n = 1)	5	Pos: 0		0	NS/Mix
		Neg: 0		0	
		NS/Mix: 1	[61]	100	
Extraversion (n = 1)	5	Pos: 0		0	NS/Mix
		Neg: 0		0	
		NS/Mix: 1	[61]	100	
Agreeableness (n = 1)	5	Pos: 0		0	NS/Mix
		Neg: 0		0	
		NS/Mix: 1	[61]	100	
Neuroticism (n = 1)	5	Pos: 0		0	NS/Mix
		Neg: 0		0	
		NS/Mix: 1	[61]	100	
Satisfaction with life (n = 1)	5	Pos: 0		0	NS/Mix
		Neg: 0		0	
		NS/Mix: 1	[61]	100	

**Variables (n ≤ 2)**	**% overall studies**	**Direction of the association (n)**	**Reference number**	**n/N (%)**	**(+, -, NS/Mix)**
Higher expectancy of control (n = 1)	5	Pos: 0	[201]	0	-
		Neg: 1		100	
		NS/Mix: 0		0	
Better model of self (n = 1)	5	Pos: 0	[152]	0	-
		Neg: 1		100	
		NS/Mix: 0		0	
Better model of others (n = 1)	5	Pos: 0	[152]	0	-
		Neg: 1		100	
		NS/Mix: 0		0	
Positive neighbourhood perception n = 1)	5	Pos: 0	[216]	0	-
		Neg: 1		100	
		NS/Mix: 0		0	
Resilience (n = 1)	5	Pos: 0	[107]	0	-
		Neg: 1		100	
		NS/Mix: 0		0	
Wisdom (n = 1)	5	Pos: 0	[90]	0	-
		Neg: 1		100	
		NS/Mix: 0		0	
Self-esteem (n = 1)	5	Pos: 0	[201]	0	-
		Neg: 1		100	
		NS/Mix:0		0	

Variables (n ≤ 2)	% overall studies	Direction of the association (n)	Reference number	n/N (%)	(+, -, NS/Mix)
Identification with the old age group (n = 1)	5	Pos: 1	[70]	100	+
		Neg: 0		0	
		NS/Mix: 0		0	
Awareness of memory problems (n = 1)	5	Pos: 1	[221]	100	+
		Neg: 0		0	
		NS/Mix: 0		0	
Interpersonal and intergroup level					
Number of known older family and friends (n = 1)	5	Pos: 0		0	NS/Mix
		Neg: 0		0	
		NS/Mix: 1	[109]	100	
Institutional and environmental level					
Available economic resources (n = 1)	5	Pos: 0		0	-
		Neg: 1	[231]	100	
		NS/Mix: 0		0	
Number of general practitioners (GPs) (n = 1)	5	Pos: 0		0	NS/Mix
		Neg: 0		0	
		NS/Mix: 1	[231]	100	
Population density (n = 1)	5	Pos: 0		0	NS/Mix
		Neg: 0		0	
		NS/Mix: 1	[231]	100	
Concentration of older population in the neighbourhood (n = 1)	5	Pos: 0		0	NS/Mix
		Neg: 0		0	
		NS/Mix: 1	[216]	100	

**Variables (n ≤ 2)**	**% overall studies**	**Direction of the association (n)**	**Reference number**	**n/N (%)**	**(+, -, NS/Mix)**
Comparative intergroup status: higher status for older (n = 1)	5	Pos: 0<br>Neg: 1<br>NS/Mix: 0	[70]	0<br>100<br>0	NS/Mix

Note: Pos – Positive association with ageism (i.e., the determinant is associated with higher ageism levels); Neg – Negative association with ageism (i.e., the determinant is associated with lower levels of ageism); NS/Mix – non-significant or mixed findings in the relation between the determinant and ageism levels; Assoc – Association; + positive association; - negative association

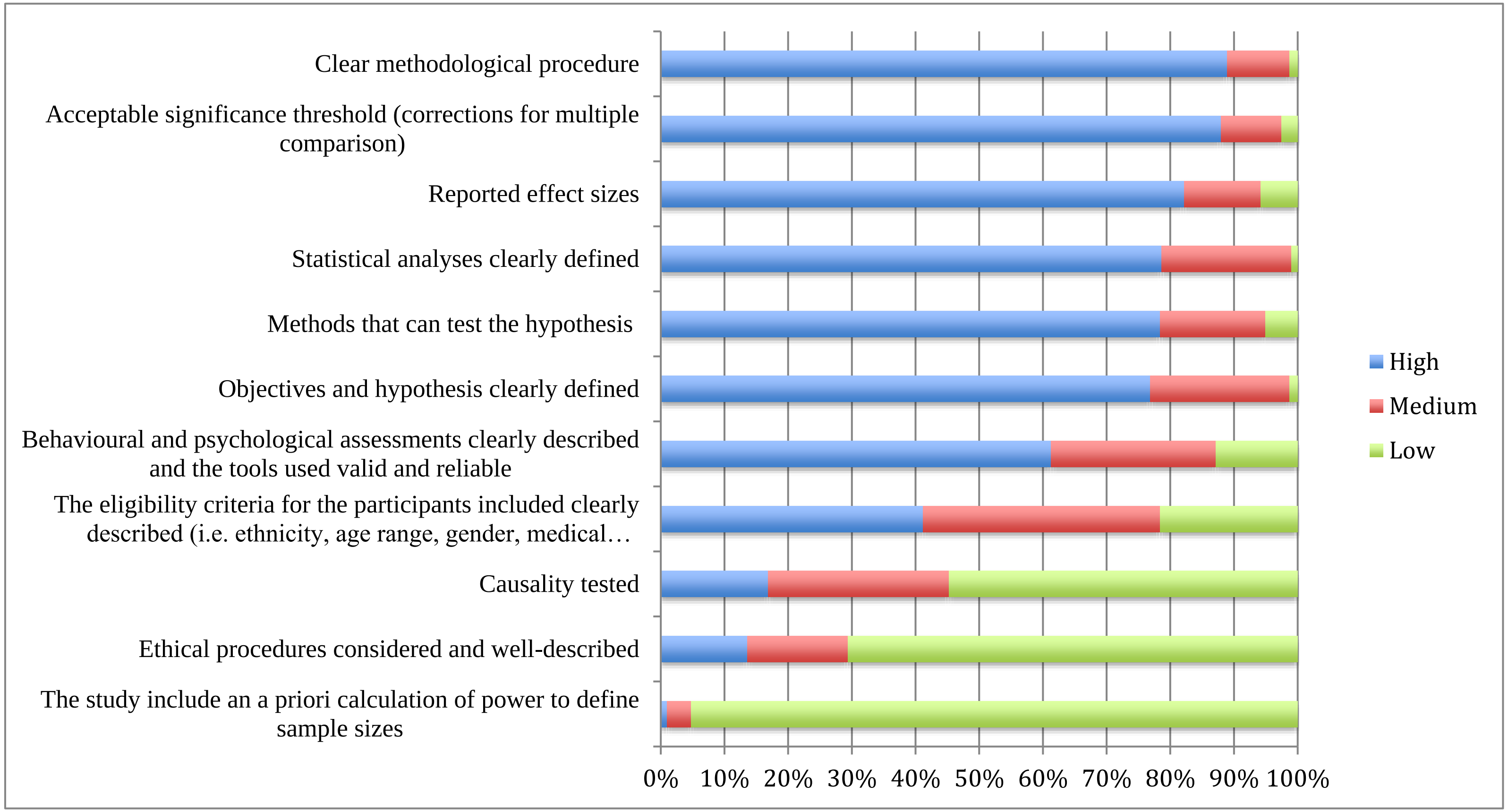

Figure S1. Summary of the quality of the studies assessment

Table S8. Quality of the evidence score

**References** (mean score for the two judges)	**Description of objectives and hypothesis**	**Methods adequacy**	**Causality**	**Description of the eligibility criteria for participants**	**Priori calculation of power effect**	**Description of validity and reliability**	**Description of the procedure**	**Description of the statistical analyses**	**Effect sizes reported (mean of extractions)**	**Definition of significance threshold set**	**Description of ethical procedures**	**%**
Adams-Price & Morse (2009)	3	2.5	2	1.5	1	2.5	3	3	3	3	1	78%
Allan et al. (2014)	3	3	1	2.5	1	3	3	3	3	3	1	81%
Ayalon (2013)	3	3	2	2.5	1	3	3	3	3	3	1	84%
Ayalon (2016)	3	3	1	3	1	3	3	3	3	3	1	82%
Bacanli et al. (1994)	2	1.5	1	2	1	3	3	2	1	1.5	1	58%
Baker (1983)	2	3	2	2	1	3	3	2.5	2.5	1.5	1	72%
Beatty (2009)	3	3	1	2.5	1	3	3	3	3	3	2	84%
Beck et al. (1979)	2.5	3	3	2	1	3	3	3	3	3	1	84%
Bell & Stanfield (1973)	3	2	2.5	2	1	3	3	2.5	2.5	3	1	78%
Bergman et al. (2013)	3	3	2.5	3	1	3	3	3	3	3	1	87%
Bhana (1983)	2	3	2.5	2	1	2	3	1.5	3	3	1	73%
Bieman-Copland & Ryan (2001)	3	3	2.5	3	1.5	3	3	3	3	3	1	88%
Bierly (1985)	3	3	1	2	1	3	3	3	1.5	3	1	75%
Bodner & Cohen-Fridel	3	2	1.5	1	1	3	3	3	3	3	3	81%
Bodner & Cohen-Friedel	3	3	1	3	1	3	3	3	3	3	2	85%
Bodner & Lazar (2008)	3	3	1	3	1	3	3	3	3	3	1.5	84%
Bodner et al. (2011)	3	3	1	3	1	3	3	3	3	3	1.5	84%
Bodner et al. (2012)	3	3	1	3	1	3	3	3	3	3	1	82%
Bodner et al. (2015)	3	3	1	2	1	3	3	3	3	3	3	85%
Boudjemadi & Gana (2012)	3	3	3	2.5	1	3	3	3	3	3	1	87%

**References** (mean score for the two judges)	**Description of objectives and hypothesis**	**Methods adequacy**	**Causality**	**Description of the eligibility criteria for participants**	**Priori calculation of power effect**	**Description of validity and reliability**	**Description of the procedure**	**Description of the statistical analyses**	**Effect sizes reported (mean of extractions)**	**Definition of significance threshold set**	**Description of ethical procedures**	**%**
Bousfield & Hutchison (2010)	2.5	3	1	2	1	2	3	3	3	3	1	75%
Bowen & Skirbekk (2013)	2	3	1	2	1	3	3	3	3	3	1	76%
Braithwaite et al. (1986)	2	3	2	3	1	3	3	2.5	1	3	1	75%
Braithwaite et al. (1993)	3	3	1	2	1	3	3	3	3	3	1	79%
Brewer & Lui (1984)	3	2	1	2.5	1	3	3	2.5	2.5	3	1	75%
Bryant et al. (2014)	3	3	1.5	3	1	3	3	3	3	3	3	90%
Burge (1978)	3	1	1	2	1	1	2	1	1	1	1	45%
Canetto et al. (1995)	3	3	1	3	1	3	3	3	3	3	1	82%
Cary et al. (2013)	3	3	1	1	1	3	3	3	3	3	2	79%
Caspi (1984)	2.5	3	2	3	1	3	3	3	3	3	1	84%
Celejewski & Dion (1998)	3	3	2	2	1	3	3	3	3	3	1	82%
Chan et al. (2012)	3	3	1	2	1	3	3	3	3	3	1	79%
Chang et al. (1984)	3	3	1	2	1	3	3	2	1	3	3	76%
Chasteen (2000)	3	3	2	2	1	3	3	3	3	3	2	85%
Chasteen (2005)	3	3	2	3	1	3	2.5	3	3	3	3	90%
Chen et al. (2017)	3	2.5	1.5	1	1	2.5	3	2	3	3	1	72%
Chen et al. (2010)	3	3	2	2	1	3	3	2.5	3	3	1	81%
Cherry et al. (2015)	3	3	1	2	1	3	3	3	3	3	3	85%
Cheung et al. (1999)	3	3	1	2	1.5	3	3	3	3	3	1	81%
Cheung et al. (2011)	3	3	2	2.5	1	3	3	3	3	3	1	84%
Chiu et al. (2001)	3	3	1	2	1	3	3	3	3	3	1	79%

**References** (mean score for the two judges)	**Description of objectives and hypothesis**	**Methods adequacy**	**Causality**	**Description of the eligibility criteria for participants**	**Priori calculation of power effect**	**Description of validity and reliability**	**Description of the procedure**	**Description of the statistical analyses**	**Effect sizes reported (mean of extractions)**	**Definition of significance threshold set**	**Description of ethical procedures**	**%**
Choi et al. (2013)	3	3	1.5	2	1	3	3	3	3	3	1	81%
Chonody & Teater (2016)	2	3	1	1	1	2.5	3	3	2.5	3	3	76%
Chopik & Giasson (2017)	3	2.5	1	3	2	2.5	2	3	3	3	1	79%
Chou & Choi (2011)	3	2.5	1.5	3	1	3	3	2.5	3	3	1	81%
Chung & Lin (2012)	2	3	2	2.5	1	3	3	3	3	3	1	81%
Clément-Guillotin et al.	2	3	3	1	1	2	3	3	3	3	1	76%
Collette-Pratt (1976)	2	2.5	1.5	1	1.5	2	3	3	2.5	3	1	70%
Connor et al. (1978)	3	3	1	2	1	3	3	3	3	3	1	79%
Cox & Barron (2012)	3	2.5	2	1.5	1.5	3	3	3	3	3	1	81%
Crew (1984)	2.5	3	1	2	1	1.5	3	3	2.5	2.5	1	70%
Cullen et al. (2009)	3	3	3	3	1	3	3	3	3	3	1	88%
DaŞBaŞ & Kesen (2015)	2	3	1	1	1	3	3	3	3	3	1	73%
Dasgupta & Greenwald	3	3	3	1	1	2	3	3	1	3	1	73%
Davidson et al. (2008)	2.5	2	2.5	2.5	1	1	2.5	2.5	2.5	2.5	1	69%
DeGuzman et al. (2014)	3	2	1	2.5	1	1.5	1.5	1.5	1	1	1.5	53%
Demir et al. (2016)	2	3	1	3	1	3	3	3	2	3	3	82%
Depaola et al. (1992)	2.5	3	2.5	2.5	1	3	3	2.5	3	3	1.5	84%
Depaola et al. (1994)	3	3	2	2.5	1	2.5	2.5	3	3	3	1.5	82%
Depaola et al. (2003)	2.5	3	2	2	1	3	3	3	3	3	2	84%
dePaulaCouto & Koller (2012)	3	3	1	2.5	1	3	3	3	3	3	3	87%
Deuisch et al. (1986)	2.5	2.5	2	2	1	1	3	2.5	1	2	2	66%

**References** (mean score for the two judges)	**Description of objectives and hypothesis**	**Methods adequacy**	**Causality**	**Description of the eligibility criteria for participants**	**Priori calculation of power effect**	**Description of validity and reliability**	**Description of the procedure**	**Description of the statistical analyses**	**Effect sizes reported (mean of extractions)**	**Definition of significance threshold set**	**Description of ethical procedures**	**%**
Diekman & Hirnisey (2007)	2.5	2	2	1	1	2	2.5	3	2	3	2	70%
Donlon et al. (2005)	3	3	1.5	3	2	3	3	3	3	3	1	87%
Drury et al. (2016)	3	3	1	2.5	1	3	3	3	3	3	1	81%
Drydakis et al. (2018)	2	2.5	2.5	2	1	2.5	3	2.5	3	3	1	76%
Duncan & Schaller (2009)	2.5	2.5	3	2.5	1	2.5	3	2.5	3	2.5	1	79%
Faulkner et al. (2007)	3	3	3	2	1	1	3	3	3	3	1	79%
Ferraro (1992)	2.5	2	1	3	1	1	2	2	2.5	3	1	64%
Finkelstein & Burke (1998)	3	3	3	1	1	2.5	3	2	3	2	1	75%
Folwell (1997)	2	2.5	2.5	1	1	3	3	3	3	3	2	79%
Freeman (2002)	3	3	1	2.5	1	3	2.5	3	2.5	3	2	81%
Fullen (2016)	3	3	1	3	3	3	3	3	3	1.5	3	90%
Fusilier & Hitt (1983)	2.5	2.5	2	1	1	2.5	3	3	3	3	2	78%
Gattuso & Saw (1998)	2.5	2.5	1.5	2.5	1	2	2.5	2.5	2.5	3	1	72%
Gattuso & Shadbolt (2002)	2.5	3	1	2	1	1	1	2	1	2	1	54%
Gekoski & Knox (1990)	3	3	3	1	1.5	1	2	2	3	3	1	72%
Gekoski et al. (1984)	2.5	3	3	1	2	2	2	2	3	2	1	72%
Gibson et al. (1993)	3	2.5	2	2	1	2	3	2	1	2	1	66%
Gluth et al. (2010)	3	3	1	1	1.5	2	3	3	3	2.5	3	79%
Gordon et al. (1988)	2	2.5	2	2	1	1	3	3	2.5	3	2	73%
Graham & Baker (1989)	2	2.5	1.5	2	1	1.5	2	3	2	2.5	1	64%
Hale (1998)	3	2	1	1.5	1	1	2.5	2.5	3	2.5	2	67%

**References** (mean score for the two judges)	**Description of objectives and hypothesis**	**Methods adequacy**	**Causality**	**Description of the eligibility criteria for participants**	**Priori calculation of power effect**	**Description of validity and reliability**	**Description of the procedure**	**Description of the statistical analyses**	**Effect sizes reported (mean of extractions)**	**Definition of significance threshold set**	**Description of ethical procedures**	**%**
Harris & Fiedler (1988)	1.5	2	1	2	1	2.5	3	2.5	3	2.5	2	70%
Harwood et al. (1994)	3	2.5	2.5	2.5	1	2.5	3	3	3	3	1	82%
Harwood et al. (2001)	2.5	2	1	1.5	1	1	2.5	3	3	2.5	1	64%
Harwood et al. (2005)	3	3	2	2.5	1	3	3	3	3	3	1	84%
Haught et al. (1999)	1.5	2.5	1	2.5	1	2	3	2.5	2.5	1.5	2	67%
Hawkins (1996)	2.5	3	1	2	1	3	2.5	3	3	2.5	1	75%
Hehman et al. (2012)	2.5	2.5	2	2	1	2.5	3	3	3	2.5	1	76%
Hertzman & Zhong (2016)	3	2	1	1.5	1	2.5	2.5	2.5	3	2.5	2.5	73%
Huang (2013)	2.5	2	1	2	1	2.5	2.5	2.5	3	2.5	1	69%
Hughes et al. (2016)	3	3	1	1.5	1	2.5	3	3	3	3	1	76%
Hummert et al. (1997)	2.5	3	1	2	1	1.5	3	3	3	2.5	1	72%
Hummert et al. (2002)	3	3	2.5	2.5	1.5	3	3	3	3	3	1.5	88%
Hummert (1993)	2.5	3	1	1.5	1	1	3	3	3	2.5	2	67%
Hummert (1994)	2.5	2.5	1.5	1.5	1	1.5	3	3	3	3	1	67%
Iweins et al. (2012)	3	3	3	1.5	1	2.5	3	3	3	2.5	1.5	82%
Jackson & Sullivan (1988)	3	2.5	1.5	2.5	1	3	3	3	3	3	1	81%
Janečková et al. (2013)	2.5	2.5	1	3	1	2	3	3	2	2.5	1.5	73%
John (2013)	3	3	1	2	1	1.5	3	2.5	3	3	1	73%
Kalavar (2001)	3	3	1.5	1.5	1	2.5	3	3	3	3	1	78%
Kane (2006)	2	2.5	1	3	1	3	3	3	3	3	1	78%
Karpinska et al. (2011)	3	3	2	2	1	1	3	3	3	3	1	76%

**References** (mean score for the two judges)	**Description of objectives and hypothesis**	**Methods adequacy**	**Causality**	**Description of the eligibility criteria for participants**	**Priori calculation of power effect**	**Description of validity and reliability**	**Description of the procedure**	**Description of the statistical analyses**	**Effect sizes reported (mean of extractions)**	**Definition of significance threshold set**	**Description of ethical procedures**	%
Katz (1990)	3	3	1	2	1	2	3	3	2.5	2.5	1	73%
Kirk (2015)	3	2	1	1.5	1	3	3	3	3	3	2	78%
Knox & Gekoski (1989)	3	3	2	1.5	1	2	3	3	3	3	1	78%
Knox et al. (1986)	2	2	1.5	2	1	3	3	3	3	3	1	75%
Kornadt & Kandler (2017)	3	2	1	3	1	2	3	3	3	3	1	76%
Kornadt & Rothermund	3	3	1	2	1	2.5	3	3	3	3	1	78%
Kornadt et al. (2013)	3	3	1	2	1	2	3	3	3	3	1	76%
Krendl (2016)	3	3	1.5	1.5	1	2.5	3	3	3	3	1	78%
Kuhlmann et al. (2017)	3	3	1	2	1	1.5	3	3	3	3	1	75%
Kulik et al. (2000)	3	3	2.5	2	1	2.5	3	3	3	3	1	82%
Kwong See & Nicoladis (2009)	3	3	2.5	3	1.5	2	3	3	3	2.5	2	87%
Laditka et al. (2011)	3	3	1.5	2.5	1	2.5	3	3	3	3	1	81%
Laidlaw et al. (2010)	2.5	3	1.5	2.5	1.5	3	3	3	3	3	2	85%
Lamont et al. (2017)	3	3	1.5	2	1	3	3	3	3	3	1	81%
Levy (1999)	2	3	1.5	3	1	3	3	3	3	3	1	81%
Levy (2008)	3	3	2.5	3	1	3	3	3	3	3	1.5	88%
Levy et al. (2015)	3	2.5	2	3	1	3	3	2.5	3	3	3	88%
Lin & Bryant (2009)	2	3	2.5	2.5	1	3	3	3	3	3	1	82%
Linville (1982)	3	3	1.5	2.5	1	3	3	3	3	3	2.5	87%
Locke-Connor & Walsh (1980)	2.5	2.5	2.5	2	1	2	2.5	3	3	3	1	72%
Löckenhoff et al. (2009)	3	3	1.5	3	1	3	3	3	3	3	1	84%

**References** (mean score for the two judges)	**Description of objectives and hypothesis**	**Methods adequacy**	**Causality**	**Description of the eligibility criteria for participants**	**Priori calculation of power effect**	**Description of validity and reliability**	**Description of the procedure**	**Description of the statistical analyses**	**Effect sizes reported (mean of extractions)**	**Definition of significance threshold set**	**Description of ethical procedures**	**%**
Lookinland & Anson (1995)	2.5	3	1	2.5	1	2.5	3	3	3	3	1	78%
Luchesi et al. (2016)	3	3	2	2.5	1	3	3	3	3	3	3	90%
Luo et al. (2013)	3	3	1.5	2.5	1	3	3	3	3	3	2	85%
Luszcz & Fitzgerald (1986)	3	3	1.5	2.5	1	3	3	3	3	3	1	82%
Lytle (2016)	3	3	2	3	1	3	3	3	3	3	1	85%
Marquet et al. (2016)	3	3	2	3	1	3	3	3	3	3	2.5	90%
Martens et al. (2004)	3	3	2.5	3	1	3	3	3	3	3	1	87%
McCann & Keaton (2013)	3	3	2	3	1	3	3	3	2.5	3	1	84%
McNamara et al. (2016)	3	3	2.5	3	1	3	3	3	3	3	1	87%
Melanson & Downe-	3	3	1.5	2.5	1	3	3	3	3	3	2.5	87%
Miller et al. (1984)	3	3	3	2.5	1	3	3	2.5	3	3	2	88%
Milligan et al. (1985)	3	3	2.5	2.5	1	2	3	3	3	3	2	85%
Milligan et al. (1989)	3	3	2.5	3	1	3	1.5	3	3	3	1.5	84%
Montepare & Zebrowitz-	3	2.5	2	3	1	3	3	3	3	3	1	84%
Narayan (2008)	2.5	3	2	2.5	1	3	2.5	3	3	3	1.5	82%
Ng et al. (2015)	3	1.5	1	2	1	2	2	2	3	3	1	66%
Nochajski et al. (2011)	3	3	1	2.5	1	3	3	3	3	3	2	84%
Nochajski et al. (2009)	2.5	1.5	1	2.5	1	2	2	2.5	3	3	2	70%
North & Fiske (2013)	3	3	3	2	1	2.5	3	3	3	3	1	84%
North & Fiske (2016)	3	3	3	3	1	2.5	3	3	3	3	1	87%
O'Connell & Rotter (1979)	3	3	1.5	3	1	3	3	3	2	3	1.5	82%

**References** (mean score for the two judges)	**Description of objectives and hypothesis**	**Methods adequacy**	**Causality**	**Description of the eligibility criteria for participants**	**Priori calculation of power effect**	**Description of validity and reliability**	**Description of the procedure**	**Description of the statistical analyses**	**Effect sizes reported (mean of extractions)**	**Definition of significance threshold set**	**Description of ethical procedures**	**%**
O'Connor & McFadden	2.5	2.5	2	1	1	1	3	2	2	3	2	67%
Obhi & Woodhead	3	3	2.5	3	1	3	3	3	3	3	3	93%
Okoye (2005)	2.5	2	1	2	1	1.5	3	2	2.5	1.5	1	61%
Oliveira et al. (2015)	2.5	3	1	3	1	2	2.5	3	2	3	3	79%
Özdemir & Bilgili (2016)	1.5	1.5	1	2	1	3	3	2	2.5	3	3	72%
Paris et al. (1997)	3	1.5	1	3	1	2	2.5	2.5	1.5	2	1	64%
Passuth & Cook (1985)	2.5	2.5	1.5	2.5	1	3	3	2.5	3	3	1	78%
Pecchioni & Croghan (2002)	3	3	1	3	1	2.5	3	2.5	3	3	1	75%
Randler et al. (2014)	2.5	1.5	1	2.5	1	2	3	2	2	2	1	63%
Reed et al. (1992)	1.5	1.5	1	2.5	1	1.5	3	2	2	3	1	61%
Revenson (1989)	2.5	3	2	2.5	1	1.5	3	2.5	3	2.5	1	75%
Rittenour & Cohen (2016)	3	3	1.5	3	1	3	3	2.5	3	3	1	82%
Roberts (2008)	3	2	1	2	2	3	3	2	2	3	1	73%
Robertson & Weiss (2017)	3	3	2.5	2	1	1.5	3	2	2.5	3	2	78%
Ruiz et al. (2015)	3	2.5	1	2.5	1	3	3	3	3	3	2	82%
Runkawatt et al. (2013)	1.5	1.5	1	2.5	1	3	3	3	3	3	3	78%
Ruscher & Hurley (2000)	3	2.5	1	1	1	1.5	3	3	3	3	1	70%
Ryan & Laurie (1990)	3	3	1.5	1.5	1	1.5	3	3	3	3	1	75%
Ryan et al. (2004)	3	3	1.5	3	1	3	2	2.5	3	3	1	79%
Sanders & Pittman (1987)	3	2	1	2	1	3	3	2.5	2	3	1	72%
Sargent-Cox et al. (2012)	3	3	1	2.5	1	2.5	3	2.5	2.5	3	1	76%

**References** (mean score for the two judges)	**Description of objectives and hypothesis**	**Methods adequacy**	**Causality**	**Description of the eligibility criteria for participants**	**Priori calculation of power effect**	**Description of validity and reliability**	**Description of the procedure**	**Description of the statistical analyses**	**Effect sizes reported (mean of extractions)**	**Definition of significance threshold set**	**Description of ethical procedures**	**%**
Sheier et al. (1978)	3	3	2.5	2	1	3	2.5	3	3	3	1	82%
Schwartz & Simmons (2001)	2.5	2	2	2	1	2.5	2	2	2	2.5	2	69%
Sherman et al. (1978)	2	3	1	1	1	1.5	2	3	3	3	1	66%
Sherman et al. (1985)	3	3	1	1.5	1	1.5	3	3	3	3	1	73%
Signori et al. (1982)	2	3	1	2	1	2	3	3	3	3	1	73%
Skorinko & Sinclair (2013)	2.5	3	3	1.5	1	3	3	3	3	3	1	82%
Smith et al. (2017)	2.5	3	1.5	2	1	3	3	3	3	3	3	85%
Soliz & Harwood (2003)	3	3	1.5	1.5	1	2	3	3	3	2.5	1	75%
Solomon & Vickers (1979)	2.5	3	1	1.5	1	2	3	2.5	2.5	3	1	70%
Springer & Harwood (2015)	3	3	2.5	1.5	1	3	3	3	3	3	1	82%
Steitz & Verner (1987)	3	3	1	1.5	1	2	2.5	2.5	3	3	1	72%
Stewart et al. (2005)	3	3	1.5	1	1	2	3	3	3	3	2	78%
Stewart & Ryan (1982)	3	3	1	1	1	2.5	3	3	3	3	2	78%
Stier & Kline (1980)	3	3	1.5	1.5	1	1.5	3	3	3	3	1	75%
Stokes & Moorman (2016)	3	3	2	2	1	1.5	3	3	3	3	2	81%
Tam et al. (2006)	3	3	1	1	1	3	3	3	3	3	1	76%
Tan et al. (2004)	3	3	1	1.5	1	1.5	3	2.5	3	3	1	72%
Thorson et al. (1974)	2.5	2.5	1.5	3	1	2	3	2	1.5	3	1	70%
Tomko & Munley (2013)	3	2.5	1	3	1	2.5	3	3	3	3	1	79%
Trigg et al. (2012)	3	3	1	3	1	3	3	3	3	3	3	88%
Turner & Crisp (2010)	3	3	3	1.5	1	2.5	3	3	3	3	2	85%

**References** (mean score for the two judges)	**Description of objectives and hypothesis**	**Methods adequacy**	**Causality**	**Description of the eligibility criteria for participants**	**Priori calculation of power effect**	**Description of validity and reliability**	**Description of the procedure**	**Description of the statistical analyses**	**Effect sizes reported (mean of extractions)**	**Definition of significance threshold set**	**Description of ethical procedures**	**%**
Vauclair et al. (2015)	3	2	1	3	1	1.5	3	2.5	2.5	2.5	1	70%
Vauclair et al. (2017)[1]	3	1.5	1	3	1	2.5	3	2	3	3	1	73%
Vauclair et al. (2017)[2]	3	1.5	1	2.5	1	2.5	3	2	2.5	3	1	70%
Verhaeghen et al. (2011)	3	3	2	3	1	2.5	3	3	3	3	1	84%
Vrugt & Schabracq (1996)	3	2.5	2	1	1	2	3	3	3	3	1	75%
Waldrop & Gress (2003)	3	3	1	3	1	3	3	3	3	3	2.5	87%
Wang et al. (2009)	3	3	1	3	1	3	3	3	3	3	2.5	87%
Wingard et al. (1982)	2.5	3	1.5	1	1	3	3	3	3	3	1	76%
Wurm et al. (2014)	3	3	1	3	1	3	3	3	3	3	2	85%
Zhang et al. (2016)	3	3	1.5	2	1	3	3	3	3	3	2	84%
Zweibel et al. (1993)	3	3	3	3	2	3	3	2	2	3	3	91%

Table S9. PRISMA checklist

Section/topic	#	Checklist item	Reported on page #
**TITLE**			
Title	1	Identify the report as a systematic review, meta-analysis, or both.	1
**ABSTRACT**			
Structured summary	2	Provide a structured summary including, as applicable: background; objectives; data sources; study eligibility criteria, participants, and interventions; study appraisal and synthesis methods; results; limitations; conclusions and implications of key findings; systematic review registration number.	1
**INTRODUCTION**			
Rationale	3	Describe the rationale for the review in the context of what is already known.	1-2
Objectives	4	Provide an explicit statement of questions being addressed with reference to participants, interventions, comparisons, outcomes, and study design (PICOS).	3
**METHODS**			
Protocol and registration	5	Indicate if a review protocol exists, if and where it can be accessed (e.g., Web address), and, if available, provide registration information including registration number.	3
Eligibility criteria	6	Specify study characteristics (e.g., PICOS, length of follow-up) and report characteristics (e.g., years considered, language, publication status) used as criteria for eligibility, giving rationale.	3; Table S1
Information sources	7	Describe all information sources (e.g., databases with dates of coverage, contact with study authors to identify additional studies) in the search and date last searched.	3
Search	8	Present full electronic search strategy for at least one database, including any limits used, such that it could be repeated.	3; Table S2
Study selection	9	State the process for selecting studies (i.e., screening, eligibility, included in systematic review, and, if applicable, included in the meta-analysis).	3

Data collection process	10	Describe method of data extraction from reports (e.g., piloted forms, independently, in duplicate) and any processes for obtaining and confirming data from investigators.	5
Data items	11	List and define all variables for which data were sought (e.g., PICOS, funding sources) and any assumptions and simplifications made.	5
Risk of bias in individual studies	12	Describe methods used for assessing risk of bias of individual studies (including specification of whether this was done at the study or outcome level), and how this information is to be used in any data synthesis.	4-5
Summary measures	13	State the principal summary measures (e.g., risk ratio, difference in means).	5-6
Synthesis of results	14	Describe the methods of handling data and combining results of studies, if done, including measures of consistency (e.g., I^2) for each meta-analysis.	5-6

**Section/topic**	**#**	**Checklist item**	**Reported on page #**
Risk of bias across studies	15	Specify any assessment of risk of bias that may affect the cumulative evidence (e.g., publication bias, selective reporting within studies).	10; Table S8
Additional analyses	16	Describe methods of additional analyses (e.g., sensitivity or subgroup analyses, meta-regression), if done, indicating which were pre-specified.	12/13
**RESULTS**			
Study selection	17	Give numbers of studies screened, assessed for eligibility, and included in the review, with reasons for exclusions at each stage, ideally with a flow diagram.	3-4; Figure 1
Study characteristics	18	For each study, present characteristics for which data were extracted (e.g., study size, PICOS, follow-up period) and provide the citations.	7-10; Table S3
Risk of bias within studies	19	Present data on risk of bias of each study and, if available, any outcome level assessment (see item 12).	Table S8
Results of individual studies	20	For all outcomes considered (benefits or harms), present, for each study: (a) simple summary data for each intervention group (b) effect estimates and confidence intervals, ideally with a forest plot.	-
Synthesis of results	21	Present results of each meta-analysis done, including confidence intervals and measures of consistency.	-
Risk of bias across studies	22	Present results of any assessment of risk of bias across studies (see Item 15).	Figure S1

Additional analysis	23	Give results of additional analyses, if done (e.g., sensitivity or subgroup analyses, meta-regression [see Item 16]).	12-13; Table 3
**DISCUSSION**			
Summary of evidence	24	Summarize the main findings including the strength of evidence for each main outcome; consider their relevance to key groups (e.g., healthcare providers, users, and policy makers).	14-15
Limitations	25	Discuss limitations at study and outcome level (e.g., risk of bias), and at review-level (e.g., incomplete retrieval of identified research, reporting bias).	15-16
Conclusions	26	Provide a general interpretation of the results in the context of other evidence, and implications for future research.	16
**FUNDING**			
Funding	27	Describe sources of funding for the systematic review and other support (e.g., supply of data); role of funders for the systematic review.	16
